# Identification and functional validation of SRC and RAPGEF1 as new direct targets of miR-203, involved in regulation of epidermal homeostasis

**DOI:** 10.1038/s41598-023-40441-w

**Published:** 2023-08-27

**Authors:** Christelle Golebiewski, Cécile Gastaldi, Diane-Lore Vieu, Bernard Mari, Roger Rezzonico, Françoise Bernerd, Claire Marionnet

**Affiliations:** 1 L’Oréal, Research and Innovation, Aulnay-Sous-Bois, France; 2 Medical Biology Department, Centre Scientifique de Monaco, Monaco, Principality of Monaco; 3 LIA BAHN, CSM-UVSQ, Monaco, Principality of Monaco; 4 Université Côte d’Azur, CNRS UMR7275, IPMC, Valbonne, France

**Keywords:** miRNAs, Physiology, Cell biology

## Abstract

The epidermis is mostly composed of keratinocytes and forms a protecting barrier against external aggressions and dehydration. Epidermal homeostasis is maintained by a fine-tuned balance between keratinocyte proliferation and differentiation. In the regulation of this process, the keratinocyte-specific miR-203 microRNA is of the outmost importance as it promotes differentiation, notably by directly targeting and down-regulating mRNA expression of genes involved in keratinocyte proliferation, such as ΔNp63, Skp2 and Msi2. We aimed at identifying new miR-203 targets involved in the regulation of keratinocyte proliferation/differentiation balance. To this end, a transcriptome analysis of human primary keratinocytes overexpressing miR-203 was performed and revealed that miR-203 overexpression inhibited functions like proliferation, mitosis and cell cycling, and activated differentiation, apoptosis and cell death. Among the down-regulated genes, 24 putative target mRNAs were identified and 8 of them were related to proliferation. We demonstrated that SRC and RAPGEF1 were direct targets of miR-203. Moreover, both were down-regulated during epidermal morphogenesis in a 3D reconstructed skin model, while miR-203 was up-regulated. Finally silencing experiments showed that SRC or RAPGEF1 contributed to keratinocyte proliferation and regulated their differentiation. Preliminary results suggest their involvement in skin carcinoma hyperproliferation. Altogether this data indicates that RAPGEF1 and SRC could be new mediators of miR-203 in epidermal homeostasis regulation.

## Introduction

MicroRNAs (miRNAs) are short (around 21–23 nucleotides) non-coding RNAs, which are able to regulate the expression of genes and are therefore involved in the modulation of diverse biological functions, such as differentiation, proliferation and apoptosis. Their regulatory action is mediated by their imperfect binding to the 3′ untranslated region (3′UTR) of target mRNAs, leading to mRNAs’ degradation or translational suppression^1, 2^. Through this mechanism, one microRNA can target hundreds of mRNAs.

Epidermal regeneration and homeostasis are essential to maintain normal skin function. The epidermis is renewed by a proliferation and differentiation process, in a continuous manner. From mitotically active basal-layer keratinocytes, some detach from the basement membrane, loose their proliferative potential and undergo a finely tuned coordinated multistep process of differentiation, to finally form the cornified layer, located at the skin surface^3, 4^. It was shown that miRNAs were of high contribution during skin morphogenesis, as their absence induced by conditionally knockout for Dicer or DGCR8 enzymes, caused severe morphological defects in mouse skin, leading to neonatal lethality by dehydration, due to cutaneous barrier impairment^5–7^.

Among skin miRNAs, miR-203 turned out to be of the outmost importance in the control of the balance between proliferation and differentiation. Identified as a skin-specific miRNA with regards to its level of expression compared to other tissues, miR-203 is one of the most abundant keratinocyte-specific miRNAs. It is specifically expressed in the suprabasal layers of the epidermis, grading from no or hardly detectable expression in the basal layer, seat of proliferative keratinocytes, to strong expression in the more differentiated suprabasal layers, suggesting a predominant involvement in keratinocyte differentiation^8–10^.

Indeed, it was shown that miR-203 induces cell-cycle exit and represses ‘‘stemness’’ in epidermal progenitor cells in vitro and in vivo in mice^9, 10^. Notably, transgenic mice overexpressing miR-203 under the control of the keratin K14 promoter—active in basal progenitors of the epidermis—exhibited a thinner epidermis and decreased proliferation. Conversely, down-regulation of miR-203 expression via antagomiR delivery resulted in an increase of epidermal cells proliferation^10^. In line with these results, the overexpression of miR-203 in human embryonic stem cells used to reconstruct in vitro pluristratified epidermis, resulted in a markedly reduced epidermal thickness^11^. In vitro, gain and loss of function assays revealed that miR-203 actually indirectly promotes differentiation by reducing clonogenic potential of mouse and human mitotically active keratinocytes, thus forcing them to exit cell cycle^9, 10, 12, 13^.

The antiproliferative function of miR-203 is exerted by the repression of direct targets in the epidermis. The first identified target of miR-203 was the transcription factor p63, in mouse^10^ and human keratinocytes^9^. Member of the p53 family, p63 has an important role in maintaining basal keratinocyte proliferative potential in normal adult epidermis^14^. In mice, two other direct targets of miR-203 have been identified Skp2 and Msi2^12^. Skp2, a transcriptional target of p63, is a cell cycle regulator promoting CDK inhibitor degradation, thus reinforcing the suppression of the p63 transcriptional network^15^. The RNA-binding protein, Msi2, fundamental regulator of cell-cycle progression through translational repression of p21/CDKN1A, was one of the drivers of the antiproliferative function of miR-203 in mouse keratinocytes. Importantly, the suppression of Skp2, p63 or Msi2 independently only partially recovered the proliferation while a simultaneous suppression of these 3 targets was required to restore almost complete proliferative capacities. These results argued that miR-203 regulation of proliferation is mediated by several targets involved in key downstream pathways, having complementary roles^12^. This is also supported by the identification in an HaCaT cell line, of other mir-203 direct targets involved in proliferation (SNAi2, LXR-α and PPAR-γ)^16, 17^.

Several other direct targets of miR-203 were identified in modified or pathological skin contexts, in which miR-203 expression is altered, with for example RAN and RAPH1 in wound healing^18^, SOCS3, IL 8, SOX6, TNF and IL24 in the context of inflammation and psoriasis^8, 19, 20^, as well as c-jun and c-myc in basal cell carcinoma (BCC) and squamous cell carcinomas (SCC) respectively^21, 22^*.*

In the context of physiological epidermal homeostasis, the understanding of the regulatory networks in which miR-203 is involved is mandatory. Therefore, this study aimed at identifying novel targets of miR-203 involved in the proliferation/differentiation balance of skin keratinocytes. The overexpression of miR-203 in human primary keratinocytes and the use of target prediction algorithms allowed us to identify new putative targets of miR-203. Luciferase assay revealed that SRC and RAPGEF1 were direct targets of miR-203 and their expression was inversely correlated to miR-203 expression during epidermal morphogenesis in a reconstructed skin model. Finally, their implication in the regulation of primary keratinocytes proliferation was demonstrated in silencing experiments.

## Results

### Determination of putative miR-203 target genes in normal human primary keratinocytes

**Figure 1:**
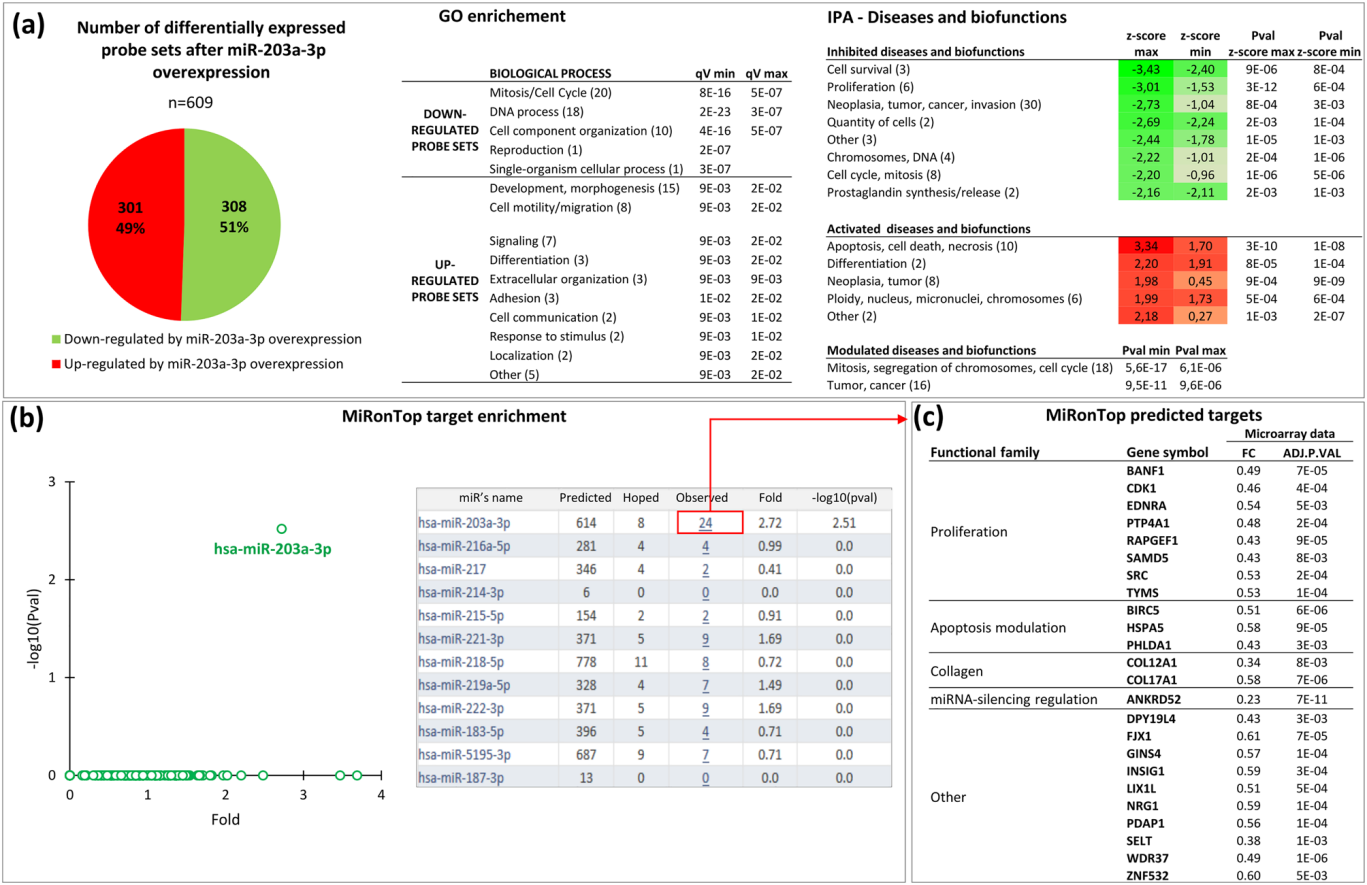
Determination of new putative miR-203a-3p targets. Normal human primary keratinocytes (NHK) were transfected with pre-miR-203 or pre-miR-control (n = 3). 30h post-transfection RNA was harvested to generate a transcriptomic profile using a full genome microarray (Agilent hsa_v2-8x60k). (**a**) Transcriptomic analysis. The criteria |log2 FC|> 0.7 and Adjusted p value < 0.05 and log2 (average expression) > 6 retrieved 609 differentially expressed probe sets between NHK overexpressing miR-203a-3p and NHK overexpressing miR-control. These differentially expressed probe sets were subjected to functional enrichment analysis with the Gene Ontology (GO) Biological Process (BP) database and Ingenuity Pathway Analysis (IPA)—Diseases and biofunctions, with |z-score|> 1.5 and pvalue < 0.05, or else pvalue < 10E−5. The tables summarized these enrichment results, with in parentheses for each pathway, the number of GO(BP) terms or of IPA Disease and Biofunctions terms (detailed lists are given in Tables S1 and S2, respectively). Minimum and maximum values of qV associated with GO enrichment, z-score and Pval z-score associated to IPA analysis, are indicated as qV min, qV max, z-score max, z-score min, Pval z-score max and Pval z-score min, respectively. (**b**) A restricted list of differentially expressed probe sets (|log2 ratio|> 0.7 and Adjpvalue < 0.01 and log2 (average expression) > 6), retrieving 174 up-regulated genes and 190 down-regulated genes, was then used to determine miR-203a-3p new targets using the bioinformatic tool MiRonTop. Representation of TargetScan predicted targets (based on their miR-203a-3p seed complementary sequences in the 3′-UTR), in the set of the 190 down-regulated genes was compared with the set of all expressed genes. A fold enrichment value and an associated *p* value were calculated. A significant enrichment of miR-203a-3p predictive targets in the set of down-regulated genes following miR-203a-3p overexpression in NHK was clearly observed. 24 potential targets were proposed. (**c**) The 24 potential targets and their described function according to literature, with their associated fold change and adjusted p value in microarray experiments, following miR-203a-3p overexpression in keratinocytes. ADJ.P.VAL, Adjusted p value; FC, fold change (pre-miR-203 transfected cells/pre-miR-control transfected cells).

To better understand how miR-203 regulates physiological epidermal homeostasis, the identification of miR-203 putative target mRNAs in normal human primary keratinocytes (NHK) was undertaken by transcriptome analysis of NHK overexpressing miR-203. Analysis of differentially expressed probe sets between NHK overexpressing miR-203 and NHK transfected with a control pre-miRNA, revealed that miR-203 overexpression modulated the expression of 609 probe sets, 308 were down-regulated and 301 were up-regulated (Fig. 1a). To identify key biological processes influenced by these differentially expressed probe sets, functional enrichment analyses were performed using Gene Ontology GO and Ingenuity Software Analysis (IPA) databases.

For down-regulated probe sets, the most significant GO terms were related to DNA process, mitosis and cell cycle, as well as cell component organization, while GO terms related to development/morphogenesis, cell motility or migration, signaling and differentiation were significantly over-represented among up-regulated probe sets (Fig. 1a and Table S1).

The functional annotation using IPA “Diseases and biofunction” database and the use of regulation z-score algorithm identified biological functions which were expected to be activated, inhibited, or modulated. The most inhibited diseases and biofunctions were related to cell survival, proliferation, cell quantity, cell cycle and mitosis, such as terms related to neoplasia, tumor, cancer, and invasion (30 terms). In turn, significantly activated diseases and biofunctions were related to apoptosis, cell death or necrosis, differentiation, and ploidy, nucleus or chromosomes (Fig. 1a and Table S2).

In summary, both enrichment analyses clearly indicated that miR-203 overexpression in human primary keratinocytes inhibited functions such as proliferation, mitosis and cell cycling; and activated differentiation, apoptosis and cell death.

Then, the extent of over-representation of specific miR-203 target mRNAs among the set of down-regulated genes was monitored using miRonTop software and TargetScan target prediction algorithm^23, 24^. A significant and clear enrichment of miR-203 predicted targets by TargetScan was observed among the genes down-regulated by miR-203 (Fig. 1b). Based on these predictions, 24 putative target mRNAs were proposed, 14 were described as involved in various functions, 8 of them were related to proliferation (Fig. 1c). Based on literature (see discussion section), although not well-described in normal human skin, we decided to focus on SRC and RAPGEF1.

### Overexpression of miR-203 in human primary keratinocytes leads to down-regulation of SRC and RAPGEF1

**Figure 2:**
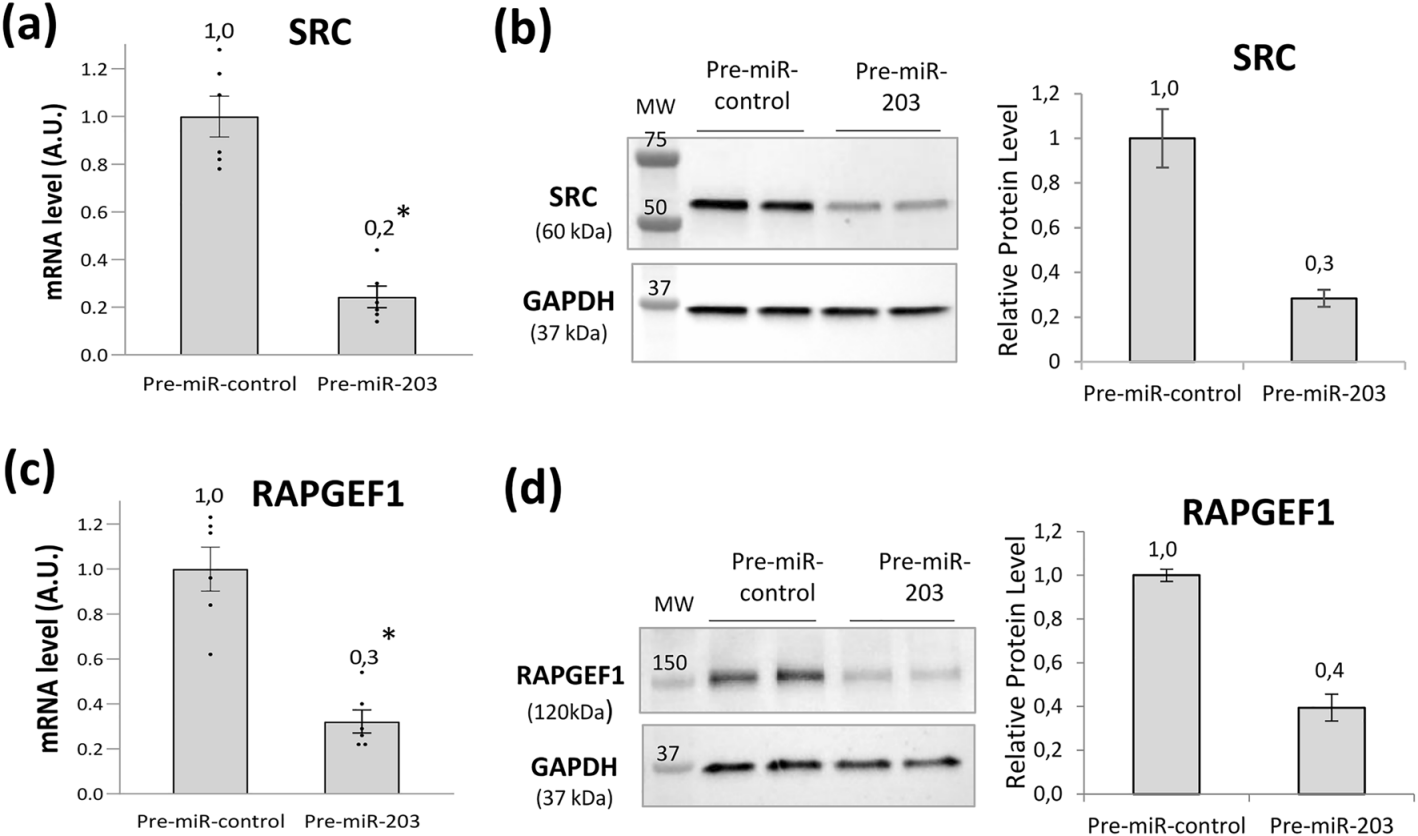
Expression of SRC and RAPGEF1 after miR-203a-3p overexpression in keratinocytes. Levels of SRC mRNA (**a**), SRC protein (**b**), RAPGEF1 mRNA (**c**) and RAPGEF1 protein (**d**) after pre-miR-203 transfection in normal human keratinocytes. mRNA levels were determined using Q-PCR 24h after pre-miR-203 transfection. Data are relative expression to RPL13A and RPS9 housekeeping genes. Histogram bars show mean value ± SEM of mRNA level. *p < 0.05, Student’s t test. Protein levels were determined by Western blot 96h after pre-miR-203 transfection. The same blot was used to detect SRC or RAPGEF1 and GAPDH proteins. Full-lengths blots are provided in Fig. S1. Signal intensity of SRC and RAPGEF1 bands were normalized using GAPDH signal intensity. Histograms show the average of normalized signal intensities of SRC and RAPGEF1. MW, molecular weight; A.U., arbitrary units.

The down-regulation assessed by microarray of the expression of SRC and RAPGEF1 mRNA after miR-203 overexpression in NHK, was confirmed using Q-PCR. 24h after pre-miR-203 transfection, SRC and RAPGEF1 mRNA expression was significantly and markedly reduced by 80% and 70%, respectively (Fig. 2a and c). 96h post-transfection of pre-miR-203, this was associated with a 70% and 60% decrease in SRC and RAPGEF1 proteins, respectively (Figs. 2b and d, S1).

### SRC and RAPGEF1 are direct targets of miR-203

**Figure 3:**
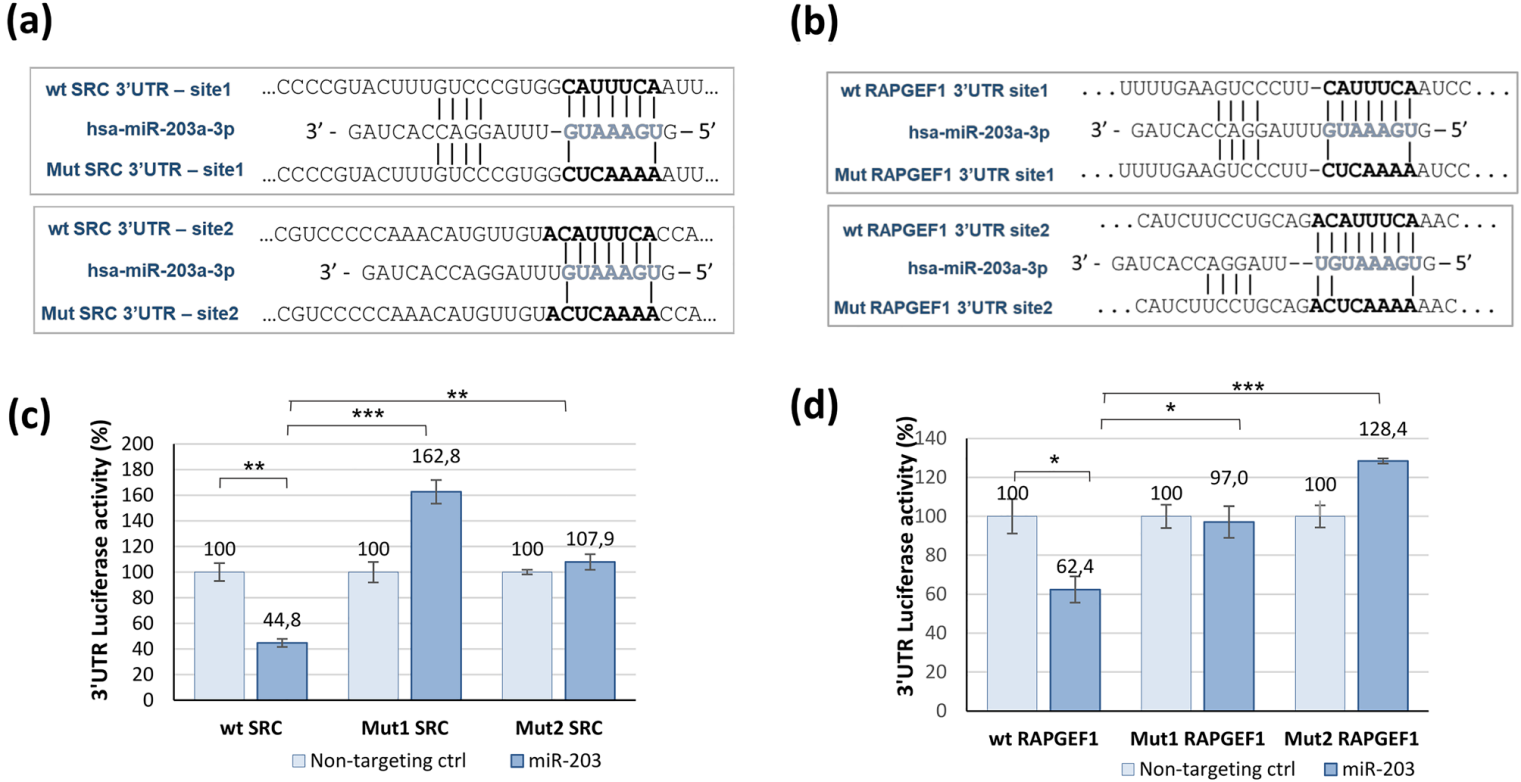
Sequences of putative binding sites of miR-203a-3p in 3′UTR sequences of SRC and RAPGEF1 and associated luciferase reporter assays of wild-type (wt) and mutated (Mut) 3′UTR. Sequences and alignments of miR-203a-3p binding sites in the 3′-UTR of SRC or RAPGEF1, and the introduced mutations in the seed sequences (**a** and **b**, respectively). The microRNA seed sequence is indicated in blue. For SRC, sites1 and 2 are at positions 1116–1122 and 1595–1601 of SRC 3′UTR, respectively. For RAPGEF1, sites1 and 2 are at positions 1832–1838 and 2665–2672 of RAPGEF1 3′UTR, respectively. Histograms for SRC (**c**) and RAPGEF1 (**d**) show the 3′UTR luciferase activity when the assay was performed in the presence of miR non-targeting control (light blue bars) or of miR-203a-3p mimic (blue bars). Data are means ± SEM of normalized luciferase activity from triplicates. *P < 0.05; **P < 0.01; ***P < 0.001 (one-way ANOVA with Dunnett’s multiple comparisons test).

TargetScan predicted two putative conserved binding sites, with a perfect base pairing in the seed region (first 2–8 bases of the mature miRNA), between miR-203 and the 3′UTR of SRC (at positions 1116–1122 and 1595–1601) and of RAPGEF1 (at positions 1832–1838 and 2665–2672) (Fig. 3a and b, respectively).

To determine if the negative regulatory effect exerted by miR-203 on SRC or RAPGEF1 expression was mediated through the direct binding of miR-203 to the identified sites in the 3′UTR of these mRNAs, a 3′UTR luciferase reporter assay was performed, using wild-type (wt) or mutated (Mut) 3′UTR.

MiR-203 overexpression significantly decreased the luciferase activity of the wt SRC 3′UTR and the wt RAPGEF1 3′UTR constructs by 55% and 38% respectively, compared to control miRNA, which did not target any human mRNA. These inhibitions were abolished after mutation of the putative binding sites, independently (Fig. 3c and d).

Altogether this data show that miR-203 decreased SRC mRNA expression by direct binding on 3′UTR of SRC, at positions 1116–1122 and 1595–1601; and decreased RAPGEF1 mRNA expression by direct binding on 3′UTR of RAPGEF1, at positions 1832–1838 and 2665–2672.

### Expression of SRC and RAPGEF1 is decreased during human primary keratinocyte differentiation

To investigate the involvement of SRC and RAPGEF1 in the epidermal proliferation/differentiation balance, their expression was examined, in parallel with miR-203, in two different models of keratinocyte differentiation (Fig. S2). When differentiation was induced by adding calcium (1.5 mM) in culture medium of NHK^25^, the miR-203 level was increased compared to culture in low calcium (50 µM). In turn, RAPGEF1 and SRC mRNA showed a slight decrease after 24h and 48h of culture (Fig. S2a). This result was more obvious in a more physiological 3D model of epidermal differentiation. An epidermis was reconstructed at the top of a dermal equivalent containing living fibroblasts, with a first phase of 7 days, during which the culture was immerged, allowing keratinocytes to proliferate and cover the dermal equivalent and a second phase of 7 days, during which the culture was emerged at the air–liquid interface, to allow keratinocyte differentiation. At the histological level and gene expression analysis, the 3D reconstructed skin allows to reproduce the major steps representative of a complete epidermal differentiation, with granular and horny layers and terminal epidermal differentiation biomarkers^26^, as well as normal features of basal keratinocytes at the dermal–epidermal junction^27^. Thus, in this model of a reconstructed epidermis (3D culture) the miR-203 level was markedly higher (×6.8) compared to the same strain of keratinocytes grown in monolayer (2D culture). Conversely, SRC and RAPGEF1 mRNA levels were down-regulated by twofold in the keratinocytes of the 3D model compared to their 2D cultured counterparts (Fig. S2b). These results show that in differentiated keratinocytes, the miR-203 level was increased, as expected, while both SRC and RAPGEF1 levels were decreased, compared to more proliferative keratinocytes.

**Figure 4:**
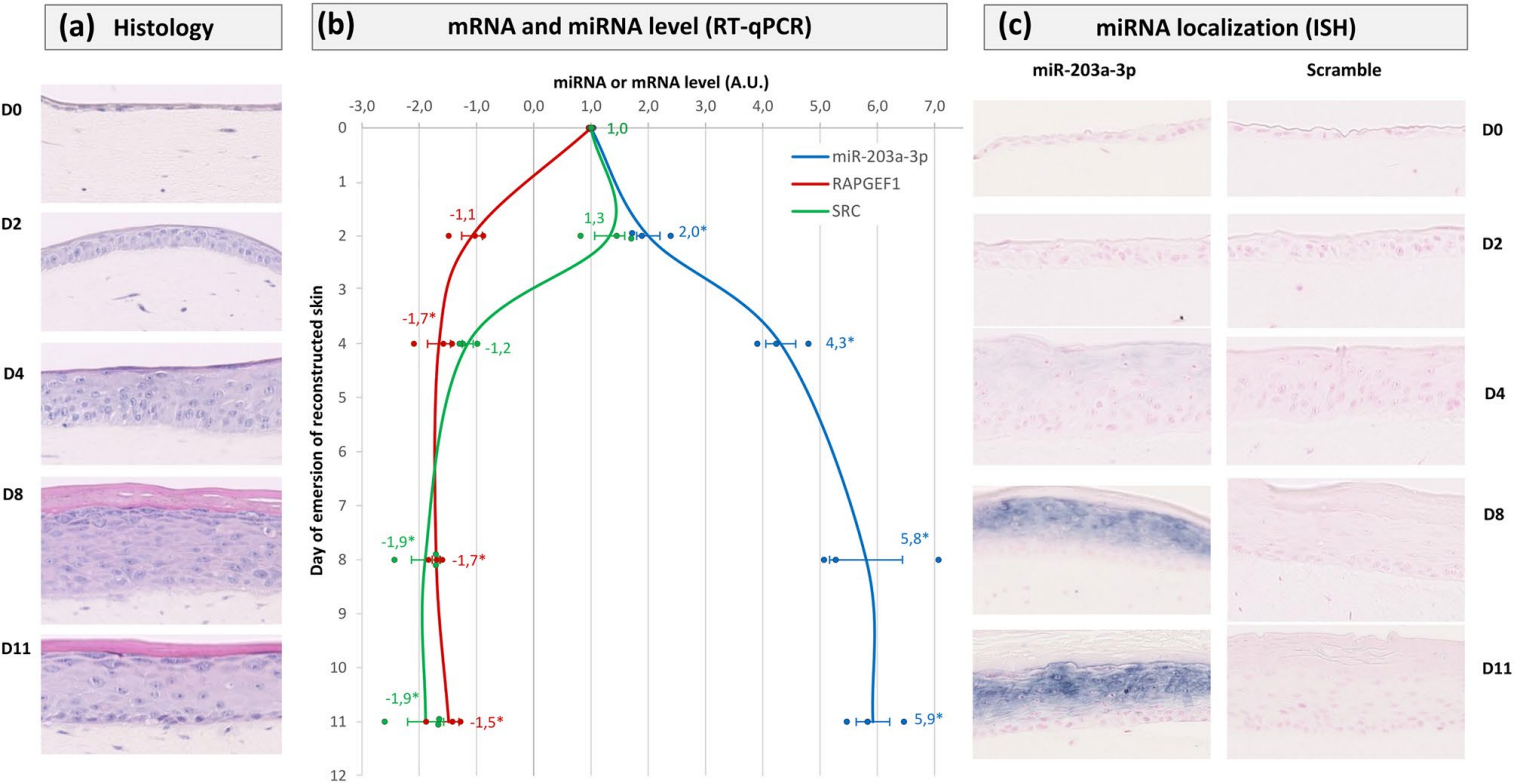
Levels of miR-203a-3p, SRC mRNA and RAPGEF1 mRNA during epidermal reconstruction. Reconstructed human skin was made of a dermal equivalent containing living fibroblasts on which NHK were seeded. The culture was kept submerged in culture medium for 7 days to allow keratinocytes to form a monolayer and then raised at the air–liquid interface (D0) up to D11, to allow keratinocytes differentiation. Samples were harvested at various time points, to perform histology analysis (**a**), RNA extraction for RT-Q-PCR to determine miR-203a-3p, SRC and RAPGEF1 mRNA levels (**b**) and in situ hybridization (ISH) of miR-203a-3p (**c**). mRNA levels were relative expression to B2M, RPL13A and RPS9 housekeeping genes. mRNA and miRNA arbitrary values were set at 1 at D0. The ratios (level at Dx)/(level at D0) were calculated for each marker. If the ratio was lower than 1 (decrease compared to D0), the ratio value was transformed into −1/Ratio. *P < 0.05, Student’s t test.

To go further into the investigation of SRC and RAPGEF1 involvement in epidermal homeostasis, their expression was assessed during epidermal reconstruction, from the first day of the emersion phase (D0) to D11, together with miR-203 expression (Fig. 4). The progressive epidermal morphogenesis was followed using histology. Starting with a monolayer of keratinocytes overlaying the dermal equivalent at D0, the differentiation process was completed, with the presence of the spinous and granular layers as well as a stratum corneum, overlaying the proliferative basal layer, at D8 (Fig. 4a). During this epidermal reconstruction, miR-203 expression was progressively increased, with a maximal expression when the epidermis was fully differentiated at D8 and maintained at D11 (Fig. 4b). At these time points, miR-203 was clearly detected in all the supra-basal compartment, using in situ hybridization (Fig. 4c). In parallel, during this reconstruction, the expression of SRC and RAPGEF1 was decreased compared to D0 with quite similar expression profiles. The SRC mRNA level was significantly decreased by twofold compared to D0, from D8 to D11. The RAPGEF1 mRNA level was significantly reduced compared to D0, from D4 to D11, by 1.5-fold on average (Fig. 4b). Altogether, these results suggest that the targets of miR-203, SRC and RAPGEF1, could be involved in the epidermal homeostasis, as their expression is modulated during epidermal reconstruction and inversely correlated with miR-203 expression. This hypothesis was next tested using functional assays.

### SRC and RAPGEF1 are involved in the proliferation/differentiation balance of human keratinocytes

The role of SRC and RAPGEF1 in epidermal homeostasis was investigated by silencing these two transcripts independently in NHK and by assessing their cellular proliferation and differentiation abilities.

**Figure 5:**
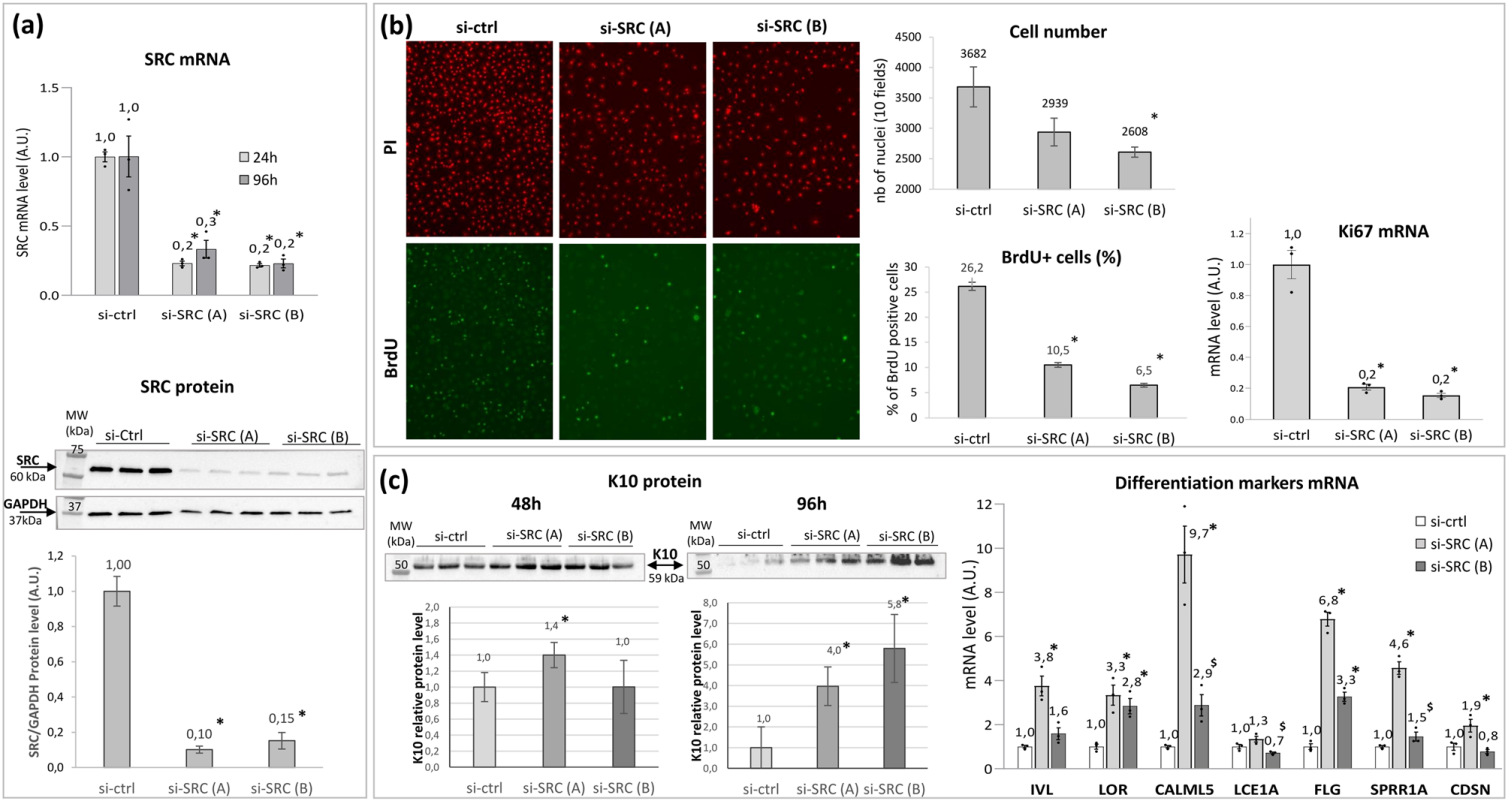
Effect of SRC silencing in human primary keratinocytes on SRC expression level (**a**), keratinocytes proliferation (**b**) and keratinocytes differentiation (**c**). Two siRNAs targeting SRC, si-SRC (A) and si-SRC (B), were independently transfected in NHK. The expression of SRC mRNA and protein was determined, at 24 and 96h using Q-PCR and at 96h post-transfection using Western Blot, respectively. The same blot was used to detect SRC and GAPDH proteins. SRC protein level was normalized using GAPDH protein level (**a**). Proliferation was assessed by BrdU assay and by measuring Ki67 mRNA expression level using Q-PCR. BrdU was incorporated 48h post-transfection; 24h later, the propidium iodide (PI) stained cells were counted and the percentage of BrdU positive cells was determined. The expression of Ki67 mRNA was quantified 96h post-transfection (**b**). Differentiation was assessed by determining the level of K10 protein using Western Blot 48 and 96h post-transfection (mean of K10 protein normalized values are shown in the histograms), and the expression level of 7 differentiation markers 96h post-transfection using Q-PCR (**c**). mRNA levels were relative expression to RPL13A and RPS9 housekeeping genes. mRNA and proteins of control conditions were adjusted to the 1 value. Histogram bars show mean value ± SEM. Significant difference vs si-ctrl in Student’s t-test is marked as *for p < 0.05 and ^§^for 0.05 < p < 0.1; A.U., arbitrary units; MW, molecular weight. Full-lengths blots are provided in Fig. S3.

NHK were transiently transfected using 2 different siRNAs targeting SRC—si-SRC (A) and si-SRC (B)—independently. SRC mRNA was significantly decreased by at least 70%, for both siRNAs, compared to a transfection using a control siRNA (si-ctrl) at 24h post-transfection. This effect was maintained until 96h. At this point, the SRC protein level was significantly reduced compared to si-ctrl, by 90% using si-SRC (A) and 85% using si-SRC (B) (Fig. 5a). In the BrdU proliferation assay, silencing of SRC reduced the number of keratinocytes in culture, in a significant manner for si-SRC (B), as revealed by the PI staining of nuclei. This decrease in cell number was due to a lower proliferation capacity of SRC-silenced keratinocytes, as attested by the significantly lower percentage of BrdU positive cells in the condition si-SRC (A) (10.5%) or si -SRC (B) (6.5%) compared to si-ctrl (26%). In addition, in NHK silenced for SRC, the expression of mRNA encoding the Ki67 proliferation marker was significantly reduced compared to control (by 80% for both siRNAs) (Fig. 5b). In turn, NHK silenced for SRC exhibited a higher expression of K10 protein (a key early marker for keratinocyte differentiation onset), with fourfold and sixfold increases 96h post-transfection of si-SRC (A) and (B), respectively (Fig. 5c). At that time point, the expression of transcripts encoding later differentiation markers (IVL, LOR, CALML5, FLG, SPRR1A and CDSN) was significantly increased when using si-SRC (A). This induction was also detected, to a lower extent, using si-SRC (B), for LOR, CALML5, FLG and SPRR1A (Fig. 5c).

**Figure 6:**
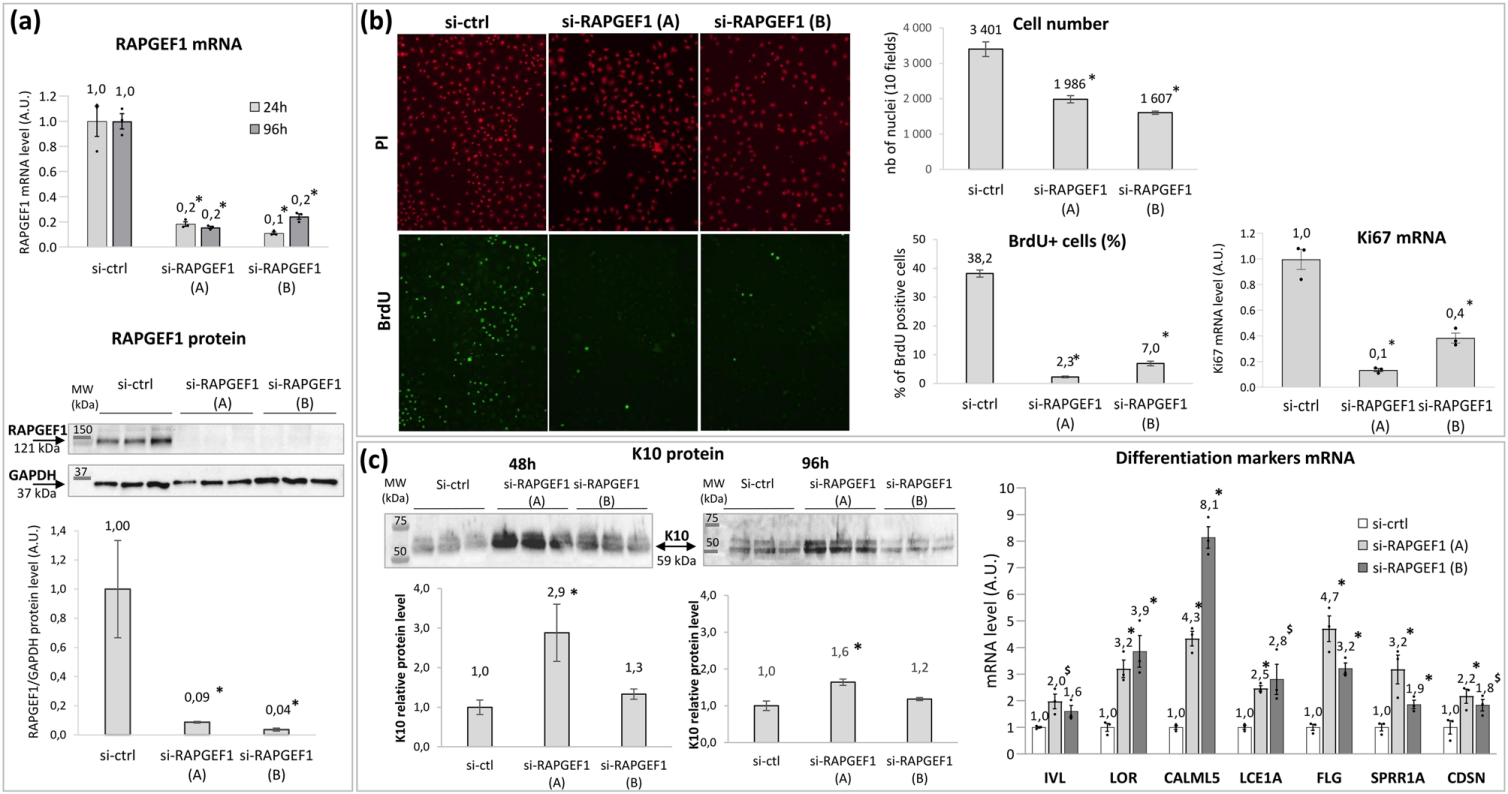
Effect of RAPGEF1 silencing in human primary keratinocytes on RAPGEF1 expression level (**a**), keratinocytes proliferation (**b**) and keratinocytes differentiation (**c**). Two siRNAs targeting RAPGEF1, si-RAPGEF1 (A) and si-RAPGEF1 (B), were independently transfected in NHK. The expression of RAPGEF1 mRNA and protein was determined, at 24 and 96h using Q-PCR and at 96h post-transfection using Western Blot, respectively. The same blot was used to detect RAPGEF1 and GAPDH proteins. RAPGEF1 protein level was normalized using GAPDH protein level (**a**). Proliferation was assessed by BrdU assay and by measuring Ki67 mRNA expression level using Q-PCR. BrdU was incorporated 48h post-transfection; 24h later, the propidium iodide (PI) stained cells were counted and the percentage of BrdU positive cells was determined. The expression of Ki67 mRNA was quantified 96h post-transfection (**b**). Differentiation was assessed by determining the level of K10 protein using Western Blot 48 and 96h post-transfection (mean of K10 protein normalized values are shown in the histograms), and the expression level of 7 differentiation markers 96h post-transfection using Q-PCR (**c**). mRNA levels were relative expression to RPL13A and RPS9 housekeeping genes. mRNA and proteins of control conditions were adjusted to the 1 value. Histogram bars show mean value ± SEM. Significant difference vs si-ctrl in Student’s t-test is marked as * for p < 0.05 and ^§^ for 0.05 < p < 0.1; A.U., arbitrary units; MW, molecular weight. Full-lengths blots are provided in Fig. S4.

The same experiments were performed to assess RAPGEF1 impact on proliferation and differentiation processes, using 2 different siRNAs targeting RAPGEF1—si-RAPGEF1 (A) and si-RAPGEF1 (B)—independently. 24 and 96h post-transfection of si-RAPGEF1 (A) or si-RAPGEF1 (B) in NHK, the RAPGEF1 mRNA level was significantly reduced by at least 80%; thus leading to a significant downregulation of RAPFEG1 protein by more than 90%, whatever the siRNA used (Fig. 6a). This RAPGFE1 silencing drastically reduced cell proliferation, as shown by the 94% and 82% decrease in BrdU-positive cells using si-RAPGEF1 (A) and si-RAPGEF1 (B), respectively, compared to transfection with the control siRNA (Fig. 6b). This decrease in proliferation led to a significant lower number of RAPGEF1-silenced NHK compared to their control counterparts. In addition, under RAPGEF1 silencing, mRNA levels of Ki67 were significantly reduced (Fig. 6b), compared to the control condition. The assessment of differentiation showed that the silencing of RAPGEF1 using si-RAPGEF1 (A) led to a significant increase in K10 protein 48h and 96h post-transfection; together with LOR, CALML5, FLG, SPRR1A mRNA expression significant increase using either si-RAPGEF1 (A) or RAPGEF1 (B), compared to control condition (p < 0.05). The use of si-RAPGEF1 (A) also significantly induced LCE1A and CDSN expression (Fig. 6c).

### Assessment of SRC and RAPGEF1 involvement in psoriasis and non-melanoma skin cancer (NMSC)

The expression of SRC and RAPGEF1 was further investigated in pathologies exhibiting keratinocyte hyperproliferation and involving miR-203, such as psoriasis and NMSC. In situ hybridizations for these transcripts were performed in 2 normal (healthy), 2 psoriatic, 2 BCC and 2 SCC skin samples.

In psoriasis, while miR-203 was strongly expressed in suprabasal layers as expected^8^, RAPGEF1 expression was restricted to epidermal basal cells, and SRC to basal and suprabasal layers excluding the uppermost layers, in mirror with miR-203 expression (Fig. S5). Compared to normal skin, the intensity of staining was higher for both, but the epidermal distribution remained unchanged.

In BCC and SCC, a strong up-regulation of SRC and RAPGEF1 was found in the tumoral zone compared to perilesional or to normal epidermis (Figs. S5a and S6a,b). In addition, using the A431 SCC cell line, we showed a strong down-regulation of miR-203 associated with a twofold up-regulation of SRC, compared to normal human keratinocytes (Fig. S6c). Conversely, overexpression of miR-203 led to the down-regulation of SRC and RAPGEF1, indicating that in an SCC cell line, miR-203 can regulate the expression of SRC and RAPGEF1 (Fig. S6d).

## Discussion

The epidermis specific miR-203 microRNA is greatly involved in the control of the balance between proliferation and differentiation of keratinocytes by directly targeting p63 transcription factor as well as Skp2 and Msi2 cell cycle regulators. In this paper, we identified SRC and RAPGEF1, as two new direct targets of miR-203, and proved their involvement in regulation of epidermal homeostasis.

To discover new putative target genes, a transcriptomic analysis was first performed in normal human keratinocytes overexpressing miR-203. Enrichment pathways analysis clearly revealed that miR-203 inhibited proliferation, mitosis and cell cycle progression and activated differentiation, apoptosis and cell death. This is in line with the transcriptomic data obtained in a metastatic SCC cells line overexpressing miR-203, in which cell cycle and cell proliferation were the most enriched pathways^22^. The major involvement of miR-203 in the regulation of pathways related to proliferation was also highlighted in an inducible miR-203 mouse skin model. The putative in vivo targets identified significantly enriched the processes of cell cycling, cell division and response to DNA damage^12^. Here we showed, in non-cancerous human keratinocytes, that miR-203 expression modulated pathways and functions which are key to maintain epidermal homeostasis.

Here, 24 putative targets for miR-203 were identified. 8 of them were described to be involved in proliferation (PTP4A1, EDNRA, BANF1, CDK1, SAMD5, TYMS, SRC protooncogene and RAPGEF1)^28–34^; 3 in apoptosis modulation (PHLDA1, BIRC5, HSPA5)^35–37^; 2 were collagens (the hemisdesmosomal keratinocyte adhesion driver COL17A1 and the wound healing-related COL12A1); 1 was the recently described regulatory subunit of the AGO2 phosphorylation cycle maintaining the global efficiency of miRNA-mediated silencing (ANKRD52)^34, 38^. The 10 other putative genes (DPY19L4, FJX1, GINS4, INSIG1, LIX1L, NRG1, PDAP1, SELT, WDR37, ZNF532) did not have to date a well-characterized function or were not precisely described in keratinocyte biology, according to literature.

Among these putative targets, some have been validated as direct targets of miR-203 in diverse cancers, such as BIRC5 in prostate and ovarian cancer cells^39, 40^, BANF1 in cervical cancer cells^41^, TYMS in colorectal cancer cells^42^ and SRC in lung and prostate cancer cells^43, 44^.

Overall, this data although found in other contexts than normal skin, shows the robustness of our results, with some miR-203 targets already described, and numerous putative targets with functions associated with miR-203 known role in epidermal homeostasis. The identified putative targets having “other”/not well-described functions may also be of interest, to get insights into miR-203’s role and mechanisms of action.

We decided to focus on SRC and RAPGEF1, as they can have a role in proliferation which is key in epidermal homeostasis, and as they are not well described in normal human skin.

Both SRC and RAPGEF1 exhibited an inverse correlation of expression with miR-203, either as miR-203 was artificially up-regulated by transfection or, more physiologically, by commitment to differentiation in a calcium switch 2D model or in a reconstructed 3D epidermis. The 3D model revealed to be the most suitable to recapitulate epidermal morphogenesis^26^. In line, Nissan et al. nicely showed in a 3D epidermal model on polycarbonate inserts, inverse profiles of expression of miR-203 (upregulation) and its DeltaNp63 target (down-regulation) during stratification and differentiation^11^.

We showed that SRC is a direct target of miR-203, through two binding sites in its 3′UTR. We confirmed the results of Wang et al., in a lung cancer cell line. They showed that mutating all the binding sequences in SRC 3′ UTR simultaneously, prevented miR-203 post-transcriptional inhibition^43^. We here show more precisely, by independent mutations, that the binding sites at positions 1116–1122 and at positions 1595–1601 of the SRC 3′UTR can drive miR-203 activity, independently.

SRC is a signal-transducing non-receptor protein kinase involved in several signaling pathways^45^. It is identified as a proto-oncogene, enriched in numerous cancers, and playing an important role in cell proliferation as well as in metastasis, cell migration and motility^46^.

In normal human skin, its role is not well-described to date. SRC is predominantly expressed in the basal layer of the human epidermis and fades when keratinocytes become more differentiated^47^ (Human Protein Atlas; https://www.proteinatlas.org/ENSG00000197122-SRC/tissue/skin and Fig. S5a), in mirror with its miR-203 regulator, which is expressed in suprabasal layers, mostly in more differentiated keratinocytes^8^ (Figs. 4 and S5a). In mice, SRC was shown to be regulated during the normal hair cycle with a specific increase during the proliferative anagen phase^7, 48^. In vitro, SRC induced human keratinocyte migration and promoted wound healing in rats^49^. Here, we showed for the first time that SRC was involved in normal human keratinocyte proliferation/differentiation balance, enhancing proliferation and inhibiting differentiation, as shown in loss of function experiments.

We also evidenced RAPGEF1 as a new target of miR-203. RAPGEF1 (also known as C3G and GRF2) is ubiquitously expressed, with high levels in adult skeletal muscle, brain, heart, kidney, lung and liver^50, 51^. It encodes a guanine nucleotide exchange factor for Ras family proteins (like Rap1, Rap2 and R-Ras), facilitating subsequent activation by the binding of GTP, leading to the activation of various signaling pathways. As such, RAPGEF1 is essential for mammalian embryonic development and cellular functions in adult tissues, such as cell proliferation, differentiation, apoptosis, and actin reorganization^34^. For example, RAPGEF1 promotes myogenic, neuronal and embryonic stem cells differentiation^52–54^ but can also either induce or inhibit proliferation^55–57^. In a context-dependent manner, it functions as an oncogene or tumor suppressor^34, 51, 58–60^. Moreover, the activation of RAPGEF1 is mainly dependent on various direct or indirect interacting proteins^34^.

RAPGEF1 is also present in the skin epidermis, preferentially located in the basal layer (Human Protein Atlas; https://www.proteinatlas.org/ENSG00000107263-RAPGEF1/tissue/skin and Fig. S5a), but its function remains unknown. Here, we showed that down-regulation of RAPGEF1 in normal human keratinocytes inhibited proliferation and promoted differentiation. Complementary experiments are needed to decipher the molecular pathways leading to the regulation of keratinocyte proliferation/differentiation. Notably, the identification of RAPGEF1 partners would be of interest. Interestingly, SRC is one of the possible proteins interacting with RAPGEF1. Their interaction leads to the activation of signaling pathways involved, for example, in cell adhesion in the MDCK epithelial cell line^61^, in focal adhesion signaling in mouse embryonic fibroblasts^62^ or in cell growth regulation in fibroblasts^63, 64^. Of note, these adhesion and cell growth functions are key in epidermal homeostasis.

To date, very little information on the regulation of RAPGEF1 expression is available. In silico analysis revealed that the promoter region contains binding sites for multiple transcription factors which have to be experimentally validated. A role of methylation of upstream regulatory sequences in RAPGEF1 expression is still a matter of debate^34, 56, 65^. Here, we showed a role of RNA interference in the regulation of RAPGEF1: miR-203 is a negative regulator of RAPGEF1 expression by binding at positions 1832–1838 and 2665–2672 in the RAPGEF1 3′UTR, thus leading to the down-regulation of RAPGEF1 mRNA expression.

As miR-203 is also involved in skin diseases where keratinocytes are hyperproliferating, such as psoriasis and NMSC, BCC and SCC, this raised the question of the role of SRC and RAPGEF1 in these pathologies. Indeed, the presence of a functional target site is not sufficient for regulation. It may be active under certain conditions but nonfunctional in a different context^66, 67^.

Psoriasis is a complex chronic inflammatory skin disease associated with a subsequent keratinocyte hyperproliferation^68^. In this pathology, miR-203 is overexpressed and displays other functions than decreasing keratinocyte proliferation. By directly down-regulating suppressor of cytokine signaling 3 (SOCS-3), miR-203 activates the STAT3 pathway and contributes to increased/prolonged skin inflammation in response to T cell-derived cytokines in psoriatic lesions^8^. SOCS-3 can also inhibit keratinocyte proliferation, thus its miR-203 suppression could also contribute to hyperproliferation^69^. Therefore, in the context of psoriatic skin, miR-203 via its direct target SOCS-3 has pro-inflammatory actions and pro-proliferative functions. By studying a limited number of samples, we showed that SRC and RAPGEF1 were expressed in layers where keratinocytes still proliferate, with an expression pattern in mirror to the one of miR-203. We could then hypothesize that SRC and RAPGEF1 could participate in keratinocyte hyperproliferation in psoriasis. Notably, it was shown that Src-family tyrosine kinases were activated in psoriasis, in correlation with the degree of hyperplasia^70^. We cannot rule out the hypothesis according to which psoriatic inflammatory microenvironment could also directly influence the level of RAPGEF1 and SRC.

In BCC and SCC, for which uncontrolled keratinocyte hyperproliferation is the main feature, miR-203 is down-regulated in line with its antiproliferative function found in normal skin. It was shown to act as a tumor suppressor by directly targeting c-jun and c-myc respectively, these protooncogenes being strongly expressed in tumoral zone^21, 22^. Our data in 4 samples tends to show that SRC and RAPGEF1 were also highly overexpressed in these tumors.

These results are in line with available published transcriptomic data from in situ SCC tumors, in which SRC and RAPGEF1 expressions were slightly but significantly up-regulated compared to normal skin, and from in situ BCC tumors, in which SRC was also found significantly up-regulated^71, 72^ (data not shown). Moreover, our experiments using the A431 SCC cell line indicate that miR-203 regulates both SRC and RAPGEF1, and that its strong down-regulation compared to normal cells is associated with an up-regulation of SRC.

Altogether, this data supports the hypothesis that down-regulation of miR-203 in SCC and BCC leads to up-regulation of its direct targets SRC and RAPGEF1, which in turn could participate in keratinocyte hyperproliferation in these pathologies. In line with this, several previous studies showed that SRC could play a role in epidermal carcinogenesis: in mouse epidermis, SRC activation induces hyperplasia and hyperkeratosis and promotes tumor, malignant progression and metastasis^47, 73, 74^ while high SRC expression and activity were evidenced in human actinic keratoses hyperproliferative premalignant skin lesions^47^.

A full dedicated study using more tissue samples and deeply exploring molecular mechanisms would be of interest to fully confirm SRC and RAPGEF1’s roles in these pathologies.

## Conclusion

We identified two new direct targets of the master epidermal regulator miR-203: SRC and RAPGEF1. The downregulation of both, independently, inhibited proliferation and promoted differentiation of normal human keratinocytes, indicating that miR-203 contribution in epidermal homeostasis could be mediated by RAPGEF1 and SRC, in addition to ΔNp63, Skp2 and Msi2. According to the literature and to our preliminary results, RAPGEF1 and SRC could also be involved in hyperproliferative skin pathologies. Moreover, this study paves the way for finding other actors of epidermal homeostasis in the miR-203 pathway, with the identification of 24 putative targets, which may play complementary roles in a coordinated process to achieve a proper epidermal homeostasis.

## Methods

### Keratinocyte and fibroblast cultures

Normal human skin was obtained from surgical residues of breast reduction surgery, with the patients’ written informed consent in accordance with the Helsinki Declaration and with Article L. 1243-4 of the French Code of Public Health. Patients’ written informed consents were collected and kept by the surgeon. The authors did not participate in sample collection. The samples were anonymized before their reception. Only age, sex and anatomical site of samples were specified. Given its special nature, surgical residue is subject to specific legislation included in the French Code of Public Health (anonymity, gratuity, sanitary/safety rules, etc.). This legislation does not require prior authorization by an ethics committee for sampling or use of surgical waste. Normal epidermal human keratinocytes (NHK) were obtained and cultured as described by Rheinwald and Green on a feeder layer of Swiss 3T3 fibroblasts^75^. Human dermal fibroblasts were isolated from mammary skin explants and grown in Dulbecco’s modified Eagle’s medium (ThermoFischer Scientific, Waltham, USA). supplemented with 10% fetal calf serum (FCS).

### In vitro reconstructed skin

Dermal equivalents were prepared as previously described using 7 ml of a mixture containing 10^6^ human dermal fibroblasts and 1.5 mg/ml native bovine type I collagen (Symatèse, Lyon, France) in a 60-mm petri dish^76^. The dermal equivalents were allowed to contract for 3 days at 37 °C, 5% CO_2_. Human epidermal keratinocytes grown in primary culture (33,000/cm^2^) were seeded on this support using stainless rings. After 2h, the rings were removed and the cultures were kept submerged for 7 days, allowing the cells to form a monolayer. The culture was then raised at the air–liquid interface (D0) and kept up for 16 days. The medium was as described previously^77^ and changed three times a week. Samples were taken at various time points during the period when cultures emerged (D0–D15).

### Histology

Samples were fixed in neutral formalin. Paraffin sections were stained with haematoxylin, eosin and saffron.

### In situ hybridization

MicroRNA in situ hybridizations were done as previously described^78^. Briefly, 5 µm-thick paraffin sections were mounted on Superfrost plus glass sides in RNAse-free conditions and deparaffinized. Skin sections were pre-treated with proteinase-K and hybridization was performed with double digoxygenin-labeled hsa-miR-203a-3p probe at a final concentration of 10 nM or with double digoxygenin-labeled scramble-miRNA probe at a final concentration of 20 nM, at 58 °C for 2 × 30 mn.

After stringent washes, with SCC buffer (Saline-Sodium Citrate buffer) and DIG blocking reagent (Roche, Mannheim, Germany), slides were incubated 2 × 15 min with alkaline phosphatase-conjugated anti-Digoxigenin and developed in 4-nitro-blue tetrazolium (NBT) and 5-brom-4-chloro-3′-indolyphosphate (BCIP) substrate (Roche, Mannheim, Germany). Counterstaining was performed with Nuclear Fast Red (Vector Laboratories, Burlingname, CA). Images were obtained using NanoZoomer 2.0-HT slide scanner (Hamamatsu, Hamamatsu-city, Japan).

### Keratinocyte transfection of pre-miRNA or siRNA

Pre-miR-203 and pre-miR-control were purchased from ThermoFischer Scientific, (Waltham, USA, #AM17100 pre-miR203a-3p ID assays PM10152 and #AM17111, respectively).

SiRNAs were purchased from ThermoFischer Scientific, Waltham, USA, with the following references: control siRNA, #12935-100; si-SRC(A), #1299001 ID HSS186080; si-SRC(B), #1299001 ID HSS186081, si-RAPGEF1(A), #1299001 ID HSS104431; si-RAPGEF1(B) #1299001 ID HSS104432.

NHK were cultured on a feeder layer of Swiss 3T3 fibroblasts as described previously^75^. At 80% confluency they were trypsinized and transfected with pre-miRNA or siRNA at a final concentration of 10 nM, using lipofectamine RNAiMAX reagent (ThermoFischer Scientific, Waltham, USA) in Opti-MEM^®^ I Reduced Serum Medium (ThermoFischer Scientific, Waltham, USA). After one night of transfection, cells were grown at 37 °C, 5% CO_2_, in KGM-Gold w/o Ca^2+^, phenol red free BulletKit (Lonza, Basel, Switzerland), prepared according to the manufacturer’s protocol but without gentamicin/amphotericin B and supplemented with calcium at a final concentration of 50 µM.

### Proliferation assay and cell counting

48h post transfection of siRNA, 5-bromo-2′- deoxy-uridine (BrdU) was added to the cell culture medium for one night. 24h later, BrdU which was incorporated into DNA was revealed using a detection kit (Roche, Mannheim, Germany). Nuclei were counterstained with propidium iodide (PI) (ThermoFischer Scientific, Waltham, USA). Incubation times were 2h for anti-BrdU working solution and 1 h for Anti-mouse-Ig-fluorescein working solution. BrdU and PI-positive cells were counted on 10 fields per slide at the 10× magnification, using Histolab software (Microvision, Evry, France). Means of cell numbers were compared using a two-tailed Student’s t-test (P < 0.05).

### Total RNA extraction

NHK grown in monolayer were lysed in TRI reagent (Merck, Darmstadt, Germany).

Reconstructed skin was rinsed in Dulbecco’s phosphate-buffered saline without calcium and magnesium (ThermoFischer Scientific, Waltham, USA). Reconstructed epidermis was peeled off from the dermal equivalent using fine forceps, immediately immersed in TRI reagent (Merck, Darmstadt, Germany) and disrupted using a glass tissue grinder (DWK Life Science, Mainz, Germany).

Total RNA was then extracted from NHK or from reconstructed epidermis according to the supplier’s instructions. The quality of total RNA was analyzed using a 2100 Bioanalyzer (Agilent Technologies, Santa Clara, USA). The amount of total RNA was quantified using the Nanodrop-One (Ozyme, Saint-Cyr-l’Ecole, France).

### Quantitative RT-PCR of mRNAs

1 µg of total RNA was used for first-strand cDNA synthesis using an Advantage RT-for-PCR kit (Takara Bio, Shiga, Japan), according to the manufacturer’s instructions. Quantitative PCR was performed using the LightCycler 480 (Roche Applied Science, Indianapolis, USA) and the LightCycler-FastStart DNA Master Sybr Green kit (Roche Diagnostics, Meylan, France) as described previously^79^. Beta-2-microglobulin (B2M), ribosomal protein L13a (RPL13A), and S9 (RPS9) mRNA were quantified in each sample and used for normalization using Genorm application^80, 81^. Means of mRNA levels were compared using a two-tailed Student’s t-test (P < 0.05). Primer sequences are detailed in Table S3.

### Quantitative RT-PCR of miRNAs

50 ng of total RNA was reverse transcribed using TaqMan microARN Reverse Transcript kit (ThermoFischer Scientific, Waltham, USA). Expression of miR-203a-3p was evaluated using TaqMan MicroRNA Assays (ThermoFischer Scientific, Waltham, USA) and the LightCycler 480 detection system. Expression levels were normalized to hsa-RNU44 and hsa-RNU48 and calculated using the comparative CT method (2^−ΔΔCT^). Means of miRNA levels were compared using a two-tailed Student’s t-test (P < 0.05).

### Protein extraction

Soluble protein fraction, containing SRC, RAPGEF1 and GAPDH, was extracted in ice-cold RIPA buffer (Thermo Scientific, Courtaboeuf, France), supplemented with a protease inhibitor cocktail (SIGMA-ALDRICH, St Louis, Missouri, USA). Lysates were clarified by centrifugation at 12,000×*g* at 4 °C. Protein quantity determination was performed on protein supernatants using a BCA assay (Thermo Scientific, Courtaboeuf, France) according to the manufacturer’s instructions.

The pellet of insoluble proteins, used for K10 detection, was re-suspended in Laemli Buffer, supplemented with protease inhibitor cocktail (SIGMA-ALDRICH, St Louis, Missouri, USA). Protein solutions were boiled at 95 °C during 5 mn. Protein concentration was determined with the 660NM Assay kit (Thermo Scientific, Courtaboeuf, France).

### Western Blot analysis

10 µg of protein extract were separated on Tris–HCl 4–12% gradient SDS-PAGE gels (BIO-RAD; Marnes-la-Coquette, France) and transferred onto pre-treated nitrocellulose membrane using transblot turbo Transfer system (BIO-RAD; Marnes-la-Coquette, France). After blocking nonspecific binding sites with 5% nonfat milk, the membranes were incubated using the following primary antibodies: mouse anti-SRC (AHO1152, Invitrogen–Fisher-Scientific, Illkirch, France) diluted to 1:250; mouse anti-RAPGEF1 (sc-17840, Santa-Cru, Heidelberg, Germany) diluted to 1:250; mouse anti-GAPDH (H86504M, Meridian, Saco, Maine, USA) diluted to 1:10,000; or mouse anti-K10 (mab3230, Millipore, Saint-Quentin-en-Yveline, France) diluted to 1:2000.

The secondary antibody used was 1:1000 dilution of polyclonal goat anti-mouse Ab IgG HRP-conjugated (P0447, DAKO, Courtaboeuf, France). Stained proteins were detected using ECL Western Blotting detection system (ThermoScientific, Courtaboeuf, France). The membrane was scanned using Image DOC (Sigma, St Louis, Missouri, USA). Band intensities were quantified using ImageLab (BIO-RAD, Marnes-la-Coquette, France). Levels of GAPDH were used to normalize SRC and RAPGEF1 protein levels. Levels of total proteins were determined by In-Gel Labeling (Stain-free technology. BIO-RAD; Marnes-la-Coquette, France) and used to normalize K10 protein level.

### Transcriptomic analysis and targets research

Transcriptomes of normal human keratinocytes transfected with 10 nM pre-miR-203 or pre-miR-control for 30h were performed using Agilent hsa_v2-8x60k full genome microarrays from Agilent Technology. Three biological independent replicates were performed per condition.

#### Labeling and hybridization

Integrity of total RNA was assessed using an Agilent BioAnalyzer 2100 (Agilent Technologies) (RIN > 9). For each sample, 200 ng of total RNA were amplified and labeled with Cy3 dye using the low RNA input QuickAmp kit (ref 5190–2305, Agilent) as recommended by the supplier. After fragmentation step, 600 ng of Cy3-labeled cRNA probes were hybridized on 8x60K v2 high density SurePrint G3 gene expression human Agilent microarrays (Agilent) with respect to the manufacturer’s protocol.

#### Statistical analysis

Microarray data analyses were performed using the Bioconductor limma package. Briefly, data were normalized using the quantile method. Replicated probes were averaged after normalization and control probes removal. Statistical significance was assessed using the limma moderated t-statistic. All P-values were adjusted for multiple testing using the Benjamini–Hochberg procedure. Differentially expressed probe sets between pre-miR-203-transfected cells and pre-miR-control transfected cells were selected based on an adjusted p-value below 0.05, an absolute log2-fold change > 0.7 and a log2 (Average Expression) > 6. Experimental data and associated microarray designs are deposited in the NCBI GEO under series GSE223523.

#### Functional enrichment analyses.

Differentially expressed probe sets were subjected to two functional enrichment analysis. One was using the Gene Ontology (GO) annotation database: over-representation of GO terms was tested using the topGO Bioconductor package. The function computes FDR (False Discovery Rate) adjusted p-value in order to account for multiple testings. Enrichment analysis was performed based on Biological Processes (BP) GO Terms and considered as significant when the adjusted p-value (qV) was below or equal to 0.05.

The other enrichment analysis was performed using Ingenuity Pathway Analysis software (http://www.ingenuity.com/) in biological themes (Diseases and biofunctions) using zscore > 1.5 (“Activated diseases and biofunctions”) or < − 1.5 (“Inhibited diseases and biofunctions”) and pvalue < 0.05; or else pvalue < 10E−5 (“Modulated diseases and biofunctions”).

#### Target prediction

Target mRNAs of miR-203a-3p were predicted using MiRonTop^23^, an online java web tool (http://www.genomique.info:8443/) integrating whole transcriptome expression data and miRNA target predictions from different sources. A list of 364 regulated genes after miR-203a-3p overexpression (absolute log2-fold change > 0.7 and adjusted P value < 0.01) and TargetScan prediction software were used.

### 3′UTR luciferase assay

In this assay, a plasmid containing the wild-type (wt) or mutated (Mut) 3′UTR cloned downstream to a luciferase reporter gene was co-transfected with a mimic of miR-203a-3p or a control miRNA, which did not target any human mRNA.

The SwitchGear GoClone 3′UTR reporter vector (Active Motif, Carlsbad, CA, USA) containing or not a mutation in the miR-203a-3p putative binding site (100 ng) along with either the LightSwitch miR-203a-3p mimic (50 nM, GUGAAAUGUUUAGGACCACUAG) or the LightSwitch non-targeting control (50 nM, UCACAACCUCCUAGAAAGAGUAGA, # MIM9001) were co-transfected in a Hela cell line at 80% confluence, using 0.15 µl of Dharma-FECT DUO transfection reagent (Dharmacon). Each construct was transfected in triplicate separately with either the miR-203a-3p mimic or the nontargeting control miRNA. 24h later, Luciferase reaction was performed for 30 min in the dark, followed by luminescence reading on a plate luminometer, according to the manufacturer’s protocol (LightSwitch Luciferase assay system, Active Motif, Carlsbad, CA, USA). Luminescence values were compared using the Student’s t-test.

#### Wild type 3′UTR reporter vectors

The SwitchGear GoClone 3′UTR reporter vectors contained the 3′ UTR sequence of either SRC (pLightSwitch_3UTR #S810898), or RAPGEF1 (pLightSwitch_3UTR # S813713) or GAPDH (pLightSwitch_3UTR #32014) or ACTB (pLightSwitch_3UTR #32013).

#### Mutant 3′UTR reporter for SRC or RAPGEF1

Five or six nucleotides were mutated within a single target site in 3′UTR of SRC or RAPGEF1 reporter construct (Fig. 3, upper panels) using the QuikChange (Stratagene) modified protocol^82^.

3′UTR reporter vectors GAPDH and ACTB which contained no seed sequence for miR-203a-3p were used to normalize the data, according to the manufacturer’s protocol.

### Supplementary Information

Supplementary Information.

## Data Availability

Experimental data and associated microarray designs are deposited in the NCBI GEO under series GSE223523.
