# Supplementary material for: Identification and functional validation of SRC and RAPGEF1 as new direct targets of miR-203, involved in regulation of epidermal homeostasis
Source: Sci Rep. 2023 Aug 27;13:14006. doi: 10.1038/s41598-023-40441-w (PMC10460794; doi:10.1038/s41598-023-40441-w)
Supplement: Supplementary file 1 — Supplementary Information. [file 41598_2023_40441_MOESM1_ESM.pdf]

SUPPLEMENTARY INFORMATION

"Identification and functional validation of SRC and RAPGEF1 as new direct targets of miR-203, involved in regulation of epidermal homeostasis" - Christelle Golebiewski, Cécile Gastaldi, Diane-Lore Vieu, Bernard Mari, Roger Rezzonico, Françoise Bernerd, Claire Marionnet.

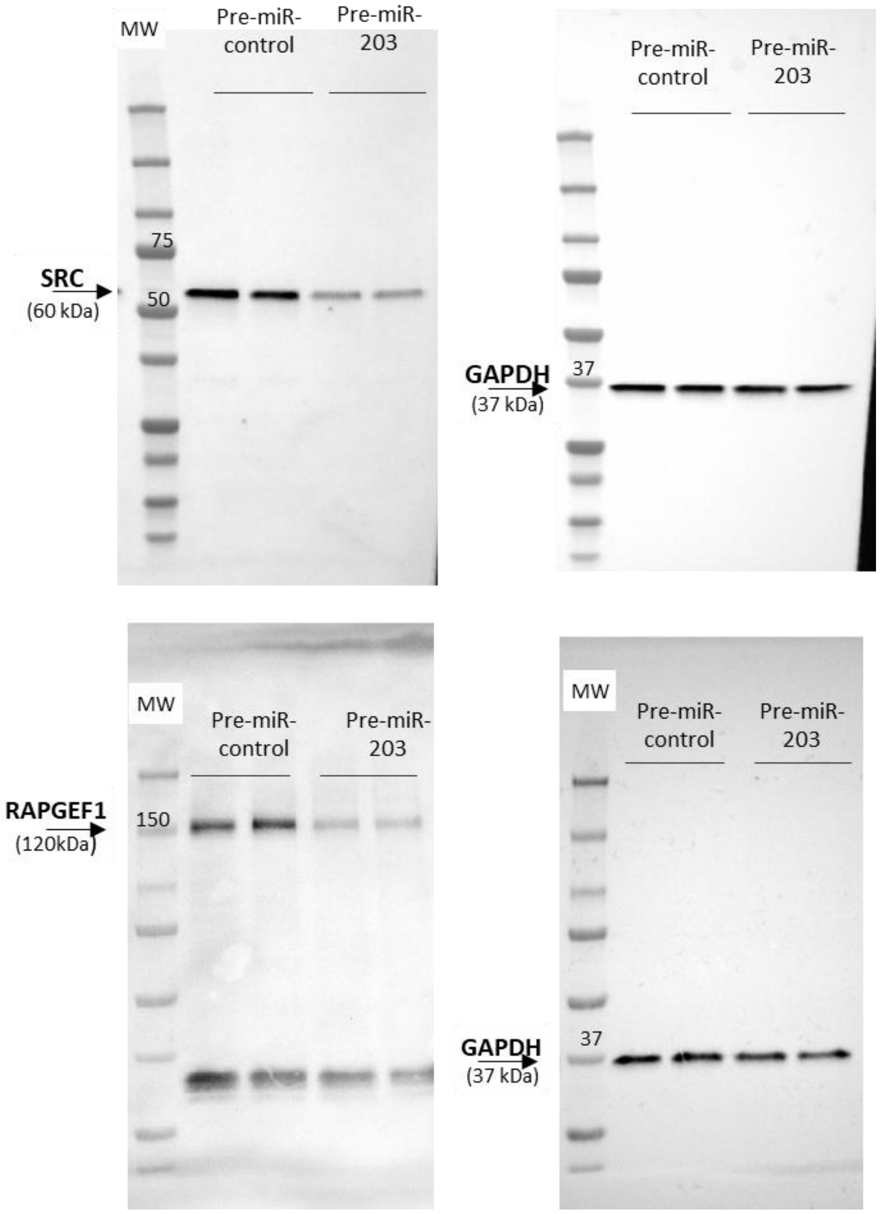

Supplementary Figure S1. Full-length images of Figure 2. The origin of these panel is identical to Figure 2 b and d, showing Western blotting with SRC, RAPGEF1 or GAPDH antibody.

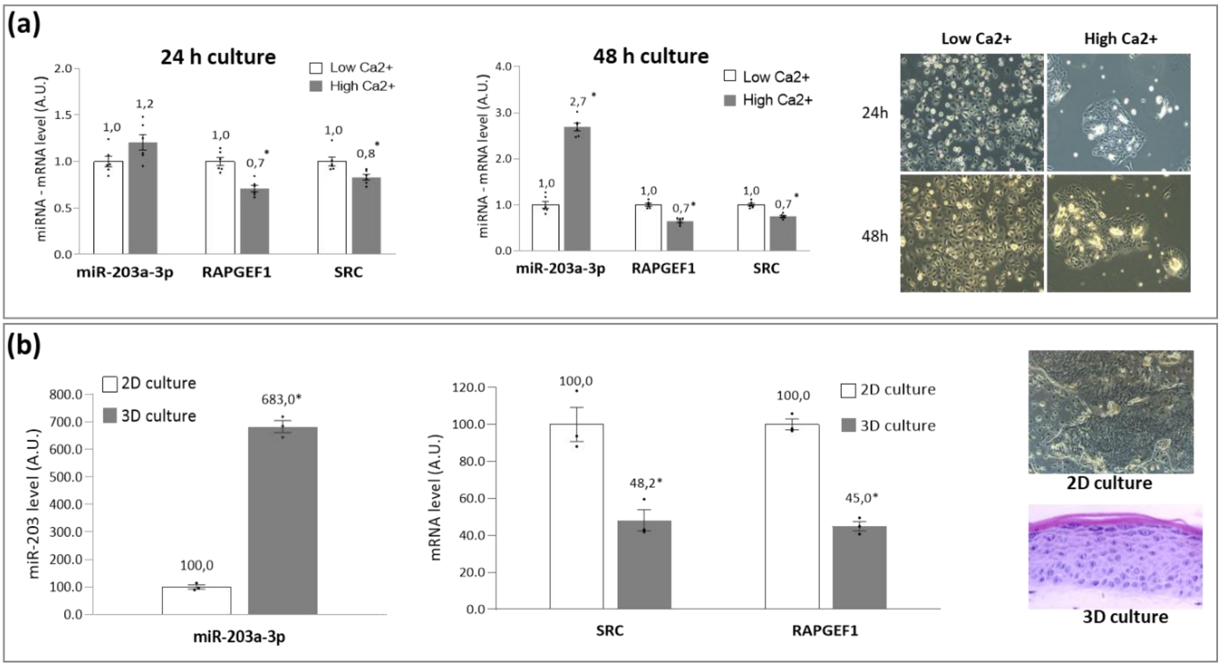

Supplementary Figure S2. Expression of SRC, RAPGEF1 and miR-203 during keratinocyte differentiation in two different culture models in vitro. **a/** Model of in vitro differentiation induced by calcium: NHK were seeded in six-well plates at a density of 2.10^5 cells per well and cultured under standard conditions in KGM2 medium containing 50µM calcium (Low Ca2+). 48h hours later differentiation was induced by increasing the calcium concentration of the growth medium to 1.5 mM (High Ca2+). 24 or 48h later, cells were harvested to extract total RNA. **b/** Model of differentiation induced by 3D culture. Normal human keratinocytes were either cultured in monolayer (2D culture), on a feeder layer of Swiss 3T3 fibroblasts as described [73], and harvested when they reached 80 % confluency; or used to reconstruct epidermis on the top of a dermal living equivalent (3D culture), and harvested 7 days post-emersion (Day 8) of the model at the air-liquid interface. In the two culture models, illustrative photographs are shown, and miRNA and mRNA levels were measured using Q-PCR. Histogram bars show mean value +/- SEM. *, different from control, p<0.05 in Student's t test. A.U., arbitrary units.

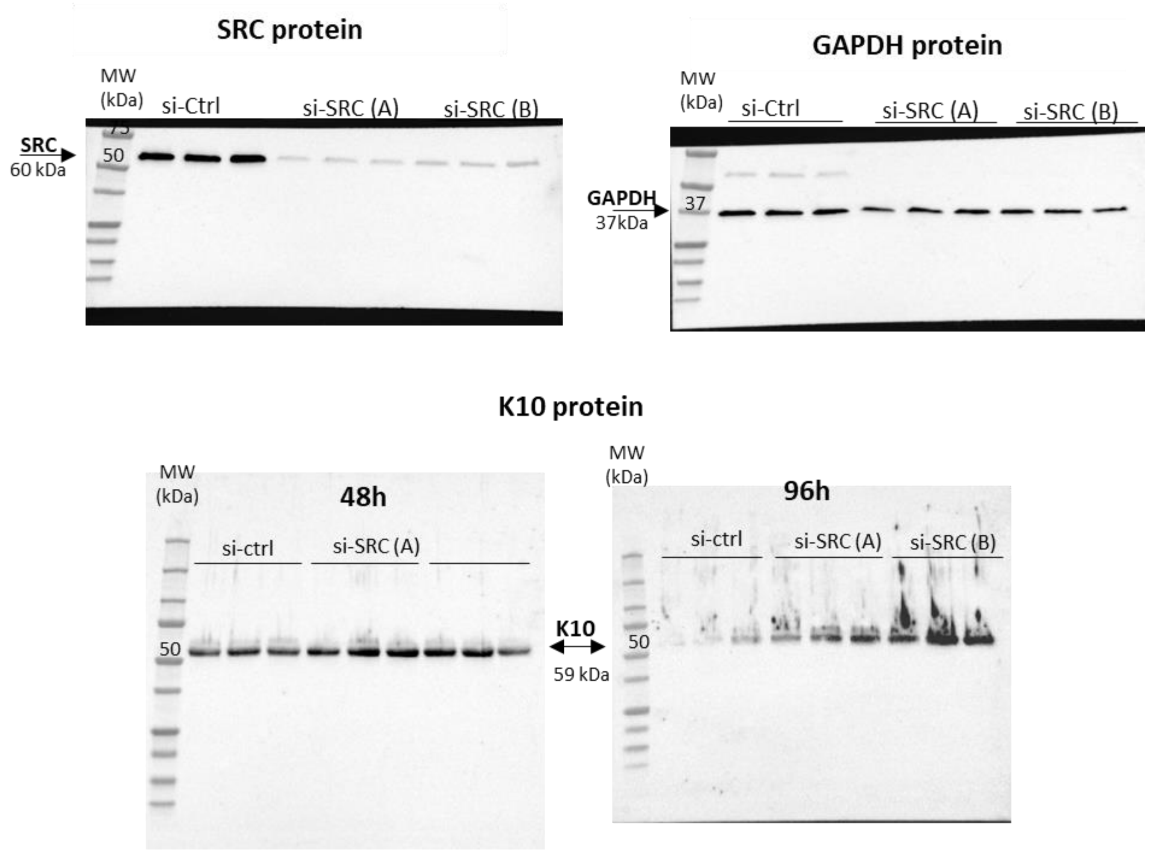

Supplementary Figure S3. Full-length images of Figure 5. The origin of these panel is identical to Figure 5 a and c, showing Western blotting with SRC, GAPDH or K10 antibody.

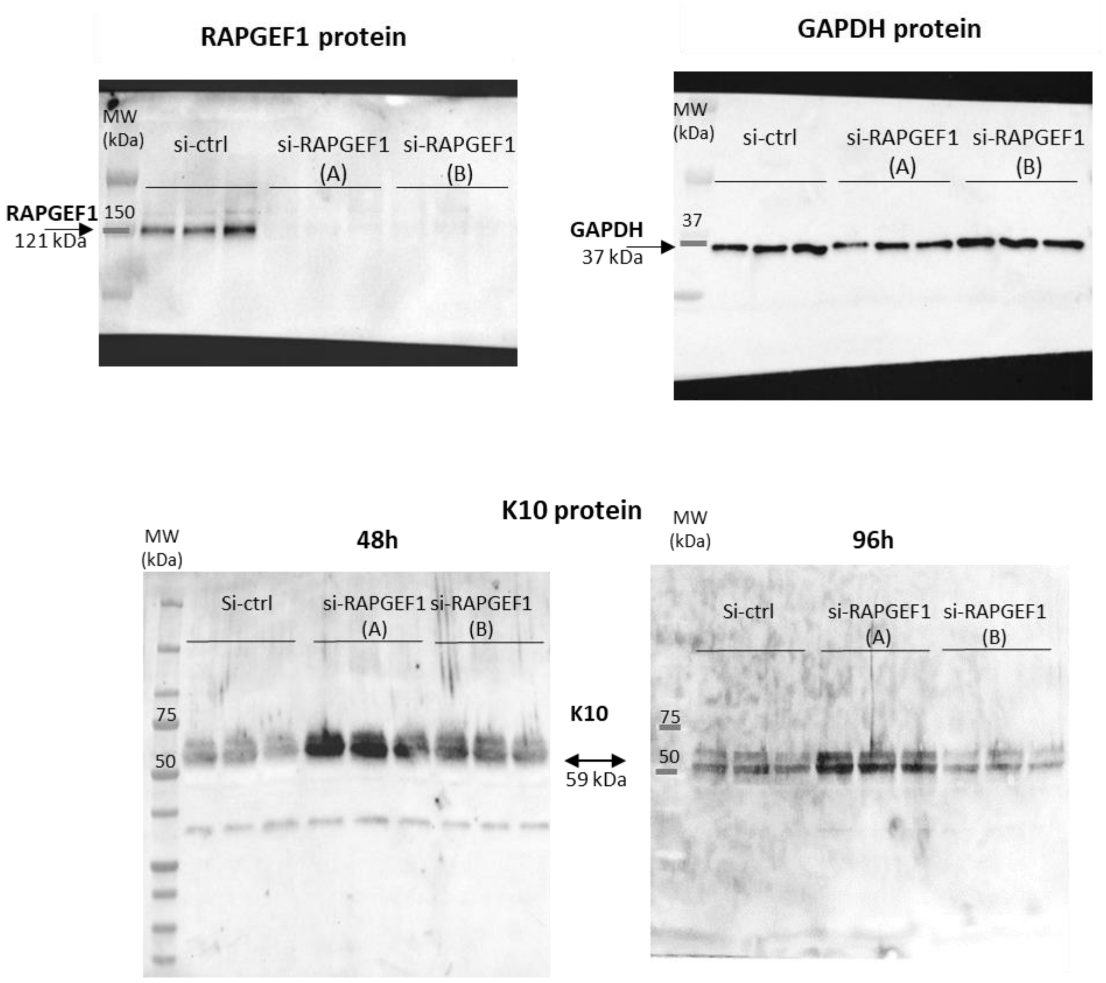

Supplementary Figure S4. Full-length images of Figure 6. The origin of these panel is identical to Figure 6 a and c, showing Western blotting with RAPGEF1, GAPDH or K10 antibody.

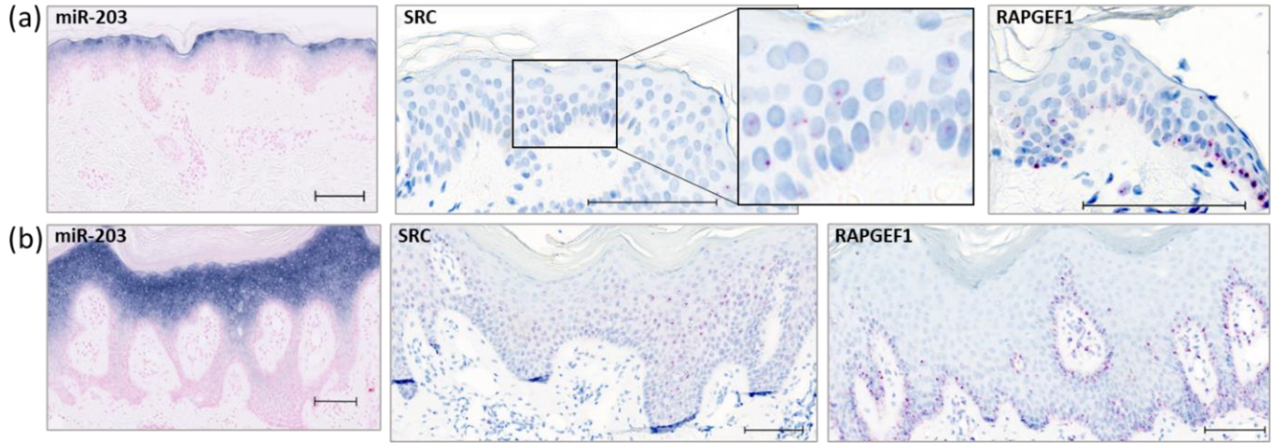

Supplementary Figure S5. Expression of miR-203, SRC and RAPGEF1 in normal (a) and psoriatic lesional skin (b) visualized by in situ hybridization.

Photographs are representative of 2 normal and 2 psoriasic skin samples. In normal human skin, while miR-203 was preferentially located in the highest suprabasal layers of the epidermis, SRC was mildly expressed in basal and first suprabasal layers and RAPGEF1 expression was restricted to basal keratinocytes. In psoriasic skin, SRC and RAPGEF1 presented the same localization than in normal skin but a higher level of expression. In parallel, miR-203 was strongly expressed in the epidermal suprabasal layers. Scale bar: 100 µm. Tissue samples were purchased from BioIVT (Burgess Hill, West Sussex, UK). mRNA in situ hybridizations were performed using Automated Chromogenic RNAscope ACD, Biotechne (Newark, CA) on 5µm-thick Formalin fixed paraffin embedded sections as described previously using the AP (red) kit at recommended experimental conditions for the Ventana Discovery Ultra instrument (Amp5 for 8 min) [Anderson et al, 2016; Møller et al. 2019]. The following RNAscope probes were obtained from ACD: SRC (NM_0055417.4, target sequence 691–2287, 20 zz pairs), RAPGEF1 (NM_001304275.2, target sequence 1199-2118, 20 zz pairs), dapB (a Bacillus subtilis gene, target sequence 414–862, 10 zz pairs). Images were acquired using a 20x objective with a Zeiss AxioScan. The negative control RNAscope probe to dapB resulted in no staining (data not shown).

Anderson et al. J. Cell. Biochem. 2016, 117, 2201–2208.

Møller et al. Int J Mol Sci 2019, doi: 10.3390/ijms20081907

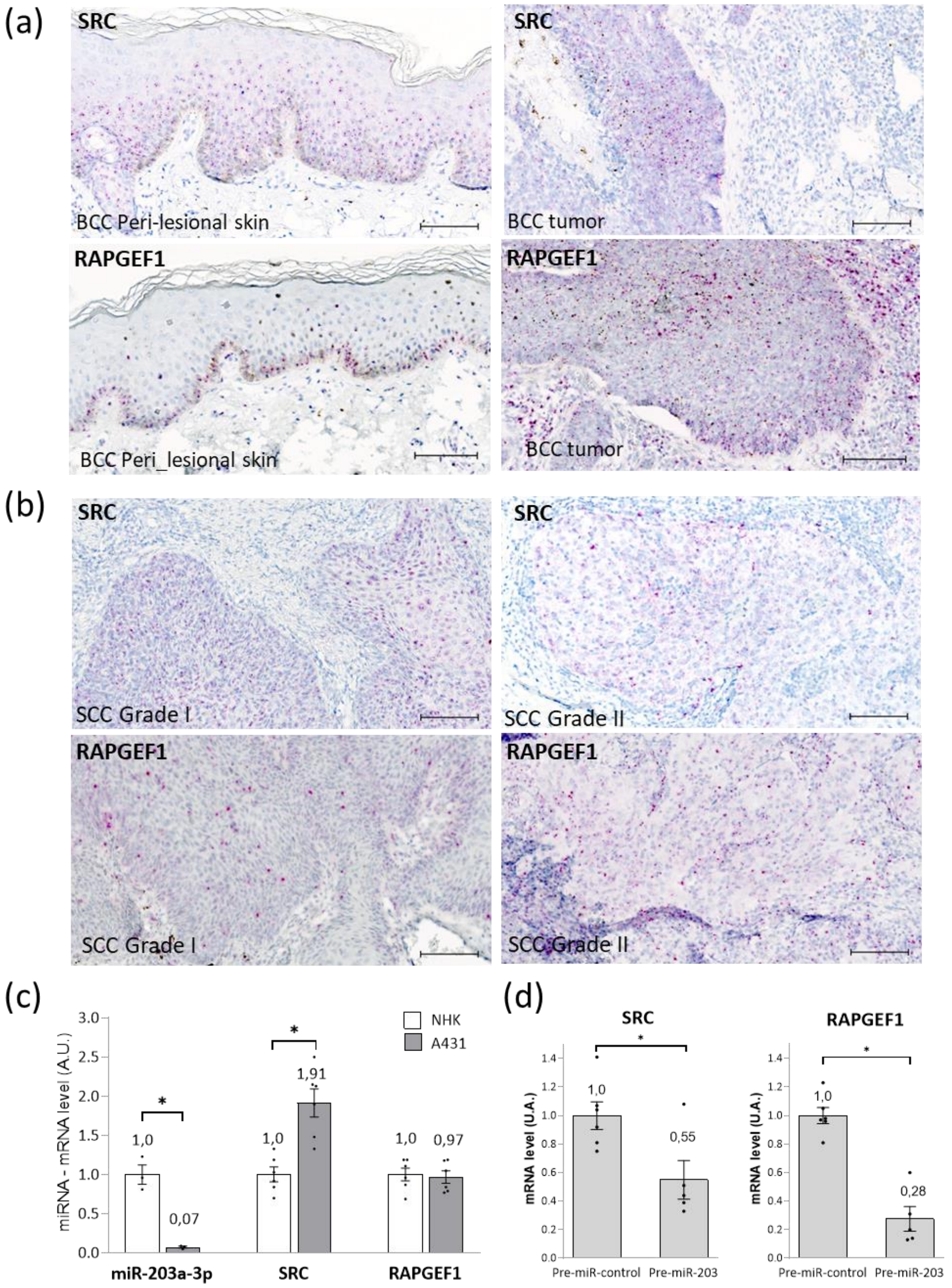

Supplementary Figure S6. Expression of SRC and RAPGEF1 in non-melanoma skin cancers. SRC and RAPGEF1 mRNA were visualized in BCC (**a**), SCC (**b**) samples, by in situ hybridization as described in Figure S5. Photographs are representative of 2 BCC, 1 Grade I SCC (well-differentiated) and 1 Grade II SCC (moderately-differentiated). Samples were purchased from BioIVT (Burgess Hill, West Sussex, UK). In BCC perilesional skin, SRC and RAPGEF1 were localized as in normal human skin, but at a higher level. In the BCC tumor their expression was found up-regulated and expressed by all the tumoral cells. This pattern was also found in the grade I and II SCCs. Scale bar: 100 µm. Level of miR-203, SRC and RAPGEF1 mRNAs in A431 SCC cell line and in normal human keratinocytes (NHK), assessed by Q-PCR (**c**). For mRNA, data are relative expression to RPL13A and RPS9 housekeeping genes. Impact of miR-203 overexpression of RAPGEG1 and SRC mRNA level in A431 SCC cell line (**d**). Levels of SRC and RAPGEF1 mRNA were determined using Q-PCR 24h after pre-miR-203 transfection. Data are relative expression to RPL13A, RPS9 and B2M housekeeping genes. Histogram bars show mean value +/- SEM of mRNA level. *p<0,05, Student's t test.

Supplementary Table S1: Most significant enriched GO terms Biological Process in primary keratinocytes overexpressing miR-203.

Up Regulated Probe Sets (301)

GOPBID		Count/Size	qV	pV
**Development, morphogenesis**				
GO:0007275	multicellular organismal development	74 / 4459	9E-03	1E-04
GO:0009653	anatomical structure morphogenesis	44 / 2319	9E-03	3E-04
GO:0009888	tissue development	36 / 1657	9E-03	1E-04
GO:0021675	nerve development	6 / 96	9E-03	1E-04
GO:0048731	system development	65 / 3891	9E-03	3E-04
GO:0060429	epithelium development	25 / 1025	9E-03	2E-04
GO:0032502	developmental process	80 / 5162	1E-02	6E-04
GO:0061448	connective tissue development	9 / 217	1E-02	9E-04
GO:0072144	glomerular mesangial cell development	2 / 4	1E-02	8E-04
GO:0044707	single-multicellular organism process	93 / 6286	1E-02	7E-04
GO:0044767	single-organism developmental process	79 / 5110	1E-02	7E-04
GO:0032501	multicellular organismal process	95 / 6510	1E-02	1E-03
GO:0007431	salivary gland development	4 / 40	1E-02	1E-03
GO:0048468	cell development	35 / 1801	1E-02	1E-03
GO:0072143	mesangial cell development	2 / 5	2E-02	1E-03
**Cell motility/migration**				
GO:0006928	cellular component movement	35 / 1613	9E-03	1E-04
GO:0016477	cell migration	25 / 1015	9E-03	2E-04
GO:0040011	locomotion	32 / 1442	9E-03	2E-04
GO:0048870	cell motility	26 / 1095	9E-03	3E-04
GO:0030334	regulation of cell migration	15 / 510	1E-02	7E-04
GO:2000145	regulation of cell motility	15 / 537	2E-02	1E-03
GO:0043537	negative regulation of blood vessel endothelial cell migration	3 / 20	2E-02	1E-03
GO:0051270	regulation of cellular component movement	16 / 604	2E-02	1E-03
**Signaling**				
GO:0009966	regulation of signal transduction	46 / 2352	9E-03	1E-04
GO:0023051	regulation of signaling	50 / 2633	9E-03	1E-04
GO:0023056	positive regulation of signaling	26 / 1111	9E-03	3E-04
GO:0009967	positive regulation of signal transduction	24 / 1054	1E-02	9E-04
GO:0014066	regulation of phosphatidylinositol 3-kinase signaling	5 / 65	1E-02	9E-04
GO:1902531	regulation of intracellular signal transduction	30 / 1442	1E-02	8E-04
GO:0007263	nitric oxide mediated signal transduction	3 / 20	2E-02	1E-03
**Differentiation**				
GO:0048710	regulation of astrocyte differentiation	4 / 24	9E-03	1E-04
GO:0030154	cell differentiation	55 / 3269	1E-02	1E-03
GO:0072008	glomerular mesangial cell differentiation	2 /5	2E-02	1E-03
**Adhesion**				
GO:0007155	cell adhesion	24 / 1054	1E-02	9E-04
GO:0022610	biological adhesion	24 / 1059	1E-02	9E-04
GO:0022408	negative regulation of cell-cell adhesion	4 / 41	2E-02	1E-03

**Extracellular organization**				
GO:0030198	extracellular matrix organization	13 / 371	9E-03	3E-04
GO:1903053	regulation of extracellular matrix organization	3 / 18	1E-02	1E-03
GO:0043062	extracellular structure organization	13 / 372	9E-03	3E-04
**Cell communication**				
GO:0010646	regulation of cell communication	50 / 2639	9E-03	1E-04
GO:0010647	positive regulation of cell communication	26 / 1116	1E-02	4E-04
**Response to stimulus**				
GO:0048583	regulation of response to stimulus	55 / 3073	9E-03	2E-04
GO:0048584	positive regulation of response to stimulus	31 / 1498	1E-02	7E-04
Localization				
GO:0051674	localization of cell	26 / 1095	9E-03	3E-04
GO:0051179	localization	77 / 5040	2E-02	1E-03
**Other**				
GO:0046903	secretion	23 / 924	9E-03	3E-04
GO:0071763	nuclear membrane organization	2 / 4	1E-02	8E-04
GO:1902932	positive regulation of alcohol biosynthetic process	3 /18	1E-02	1E-03
GO:0042063	gliogenesis	8 / 183	2E-02	1E-03
GO:0032370	positive regulation of lipid transport	4 / 43	2E-02	1E-03

Down Regulated Probe Sets (308)

GOPBID		Count/Size	qV	pV
**Mitosis/Cell Cycle**				
GO:0044772	mitotic cell cycle phase transition	37 / 472	8E-16	4E-18
GO:0044770	cell cycle phase transition	37 / 483	1E-15	8E-18
GO:0000819	sister chromatid segregation	17 / 68	3E-15	2E-17
GO:0000070	mitotic sister chromatid segregation	16 / 60	7E-15	7E-17
GO:0000075	cell cycle checkpoint	21 / 247	1E-09	2E-11
GO:0010564	regulation of cell cycle process	28 / 473	3E-09	6E-11
GO:0008608	attachment of spindle microtubules to kinetochore	8 / 21	7E-09	2E-10
GO:0051726	regulation of cell cycle	36 / 816	1E-08	4E-10
GO:0007346	regulation of mitotic cell cycle	24 / 386	2E-08	6E-10
GO:0051302	regulation of cell division	19 / 240	2E-08	7E-10
GO:0031577	spindle checkpoint	10 / 53	5E-08	2E-09
GO:0000082	G1/S transition of mitotic cell cycle	18 / 241	1E-07	5E-09
GO:0044843	cell cycle G1/S phase transition	18 / 243	1E-07	6E-09
GO:0007088	regulation of mitosis	13 / 118	2E-07	8E-09
GO:1901990	regulation of mitotic cell cycle phase transition	18 / 257	3E-07	1E-08
GO:1901987	regulation of cell cycle phase transition	18 / 266	4E-07	3E-08
GO:0007080	mitotic metaphase plate congression	6 / 15	5E-07	3E-08
GO:0007091	metaphase/anaphase transition of mitotic cell cycle	9 / 52	5E-07	3E-08
GO:0007051	spindle organization	11 / 89	5E-07	3E-08
GO:0044784	metaphase/anaphase transition of cell cycle	9 / 53	5E-07	4E-08
**DNA process**				
GO:0007059	chromosome segregation	31 / 169	2E-23	2E-26
GO:0006259	DNA metabolic process	54 / 928	9E-18	2E-20

GO:0051276	chromosome organization	42 / 811	3E-12	5E-14
GO:0006260	DNA replication	26 / 306	5E-12	8E-14
GO:0006261	DNA-dependent DNA replication	18 / 127	7E-12	1E-13
GO:0071103	DNA conformation change	20 / 201	2E-10	4E-12
GO:0006323	DNA packaging	16 / 145	5E-09	1E-10
GO:0006271	DNA strand elongation involved in DNA replication	9 / 34	2E-08	5E-10
GO:0034508	centromere complex assembly	9 / 35	2E-08	7E-10
GO:0031055	chromatin remodeling at centromere	8 / 25	3E-08	9E-10
GO:0022616	DNA strand elongation	9 / 37	3E-08	1E-09
GO:0051303	establishment of chromosome localization	8 / 27	5E-08	2E-09
GO:0050000	chromosome localization	8 / 29	9E-08	3E-09
GO:0006336	DNA replication-independent nucleosome assembly	8 / 33	2E-07	1E-08
GO:0034724	DNA replication-independent nucleosome organization	8 / 33	2E-07	1E-08
GO:0034080	CENP-A containing nucleosome assembly	7 / 23	3E-07	2E-08
GO:0061641	CENP-A containing chromatin organization	7 / 23	3E-07	2E-08
GO:0065004	protein-DNA complex assembly	13 / 127	3E-07	2E-08
**Cell component organization**				
GO:0006996	organelle organization	91 / 2685	4E-16	2E-18
GO:1902589	single-organism organelle organization	71 / 1790	2E-15	2E-17
GO:0000226	microtubule cytoskeleton organization	25 / 333	2E-10	4E-12
GO:0016043	cellular component organization	122 / 4923	6E-13	7E-15
GO:0071840	cellular component organization or biogenesis	122 / 5027	3E-12	4E-14
GO:0007017	microtubule-based process	30 / 511	7E-10	1E-11
GO:0043933	macromolecular complex subunit organization	50 / 1592	3E-07	1E-08
GO:0071822	protein complex subunit organization	46 / 1419	4E-07	2E-08
GO:0032507	maintenance of protein location in cell	12 / 107	4E-07	2E-08
GO:0034453	microtubule anchoring	8 / 38	5E-07	4E-08
**Reproduction**				
GO:0000003	reproduction	36 / 925	2E-07	1E-08
**Single-organism cellular process**				
GO:0044763	single-organism cellular process	192 / 11582	3E-07	1E-08

Supplementary Table S1. Most significant enriched GO terms Biological Process in primary keratinocytes overexpressing miR-203. Detailed list of the top 50 enriched GO terms related to Biological Process (BP) for the 301 up-regulated probe sets and 308 down-regulated probe sets in primary keratinocytes overexpressing miR-203. GOBPID: Gene ontology identity of enriched terms. Count is the number of differentially expressed probe sets on microarray belonging to specific GO identities. Size is the total number of probes on microarray belonging to specific GO identities. pv, p-value: qv, adjusted pvalue.

Supplementary Table S2: Enriched Ingenuity Pathway Analysis IPA - Diseases and Biofunctions terms in primary keratinocytes overexpressing miR-203.

Activation

	Diseases and Bio Functions	z-scores	Pval
apoptosis, cell death, necrosis	apoptosis	3,34	3E-10
	apoptosis of cervical cancer cell lines	3,21	5E-06
	cell death	2,75	1E-08
	cell death of cervical cancer cell lines	2,47	1E-06
	organismal death	2,23	7E-07
	necrosis	2,23	4E-07
	death of embryo	2,14	4E-04
	morbidity or mortality	2,01	5E-07
	apoptosis of tumor cell lines	2,01	2E-08
	cell death of tumor cell lines	1,70	1E-08
differentiation	differentiation of lymphatic system cells	2,20	8E-05
	differentiation of bone marrow cells	1,91	1E-04
neoplasia, tumor	papilloma	1,98	9E-04
	adenoma	1,95	2E-05
	benign neoplasia	1,91	8E-13
	mammary tumor	1,36	6E-08
	adenocarcinoma	1,14	5E-06
	urogenital cancer	1,07	3E-06
	tumorigenesis of tissue	0,63	1E-08
	neoplasia of epithelial tissue	0,45	9E-09
ploidy, nucleus, micronuclei, chromosomes	missegregation of chromosomes	1,99	5E-04
	formation of micronuclei	1,98	4E-04
	polyploidy of cells	1,95	4E-04
	polyploidy	1,95	5E-04
	ploidy	1,73	2E-05
	formation of nucleus	1,73	6E-04
other	formation of mitotic spindle	2,18	1E-03
	injury of mice	0,27	2E-07

Inhibition

	Diseases and Bio Functions	z-scores	Pval
proliferation	proliferation of cells	-3,01	3E-12
	cell proliferation of tumor cell lines	-2,97	2E-09
	cell proliferation of breast cancer cell lines	-2,30	9E-04
	proliferation of myeloid cells	-2,21	3E-03
	cell proliferation of carcinoma cell lines	-2,18	3E-04
	cell proliferation of squamous cell carcinoma cell lines	-1,53	6E-04
cell cycle, mitosis	G1/S phase transition	-2,20	1E-06

	G1 phase	-2,20	7E-06
	M phase	-1,91	5E-10
	interphase of tumor cell lines	-1,78	3E-06
	M phase of tumor cell lines	-1,23	1E-08
	interphase	-1,04	4E-09
	mitotic exit	-1,00	1E-05
	G2 phase	-0,96	5E-06
neoplasia, tumor, cancer, invasion	cell viability of cervical cancer cell lines	-2,73	8E-04
	cell proliferation of colorectal cancer cell lines	-2,69	1E-05
	cell viability of tumor cell lines	-2,52	7E-06
	digestive system cancer	-2,45	1E-07
	gastrointestinal tract cancer	-2,35	7E-08
	thoracic neoplasm	-2,28	3E-05
	lung tumor	-2,22	3E-05
	advanced stage solid tumor	-2,17	1E-04
	metastatic solid tumor	-2,17	2E-03
	invasion of tumor cell lines	-2,04	1E-04
	abdominal cancer	-1,96	9E-08
	invasion of cells	-1,95	2E-06
	invasion of breast cancer cell lines	-1,94	2E-04
	colorectal neoplasia	-1,91	7E-09
	large intestine neoplasm	-1,91	6E-07
	malignant solid tumor	-1,76	6E-08
	digestive organ tumor	-1,67	2E-07
	thoracic cancer	-1,67	7E-05
	colorectal cancer	-1,66	3E-08
	malignant neoplasm of large intestine	-1,66	9E-07
	liver cancer	-1,66	3E-03
	growth of malignant tumor	-1,60	4E-04
	lung cancer	-1,58	7E-05
	cell viability of carcinoma cell lines	-1,54	9E-04
	solid tumor of head and neck	-1,51	1E-03
	Gastrointestinal Tract Cancer and Tumors	-1,50	5E-08
	abdominal neoplasm	-1,43	9E-08
	tumorigenesis of genital organ	-1,07	2E-07
	genital tumor	-1,07	2E-07
	neuroepithelial tumor	-1,04	9E-06
chromosomes, DNA	synthesis of DNA	-2,22	2E-04
	alignment of chromosomes	-2,11	5E-09
	chromosomal congression of chromosomes	-1,34	1E-07
	metabolism of DNA	-1,01	1E-06
cell survival	cell survival	-3,43	9E-06
	cell viability	-3,32	6E-05
	survival of organism	-2,40	8E-04
	release of prostaglandin	-2,16	2E-03

prostaglandin synthesis/release	synthesis of prostaglandin D2	-2,11	1E-03
quantity of cells	quantity of blood cells	-2,69	2E-03
	quantity of cells	-2,24	1E-04
other	cytokinesis	-2,44	1E-05
	activation of epithelial cells	-1,98	3E-03
	microtubule dynamics	-1,78	1E-03

Modulation

	Diseases and Bio Functions	z-scores	Pval
mitosis, segregation of chromosomes, cell cycle	mitosis of cervical cancer cell lines		6E-17
	segregation of chromosomes		5E-16
	arrest in mitosis		2E-14
	arrest in mitosis of tumor cell lines		2E-11
	delay in mitosis of tumor cell lines		1E-10
	arrest in mitosis of cervical cancer cell lines		4E-10
	delay in mitosis of cervical cancer cell lines		7E-10
	attachment of kinetochores		7E-10
	attachment of spindle fibers		1E-07
	segregation of sister chromatids		4E-07
	arrest in interphase		5E-07
	arrest in M phase		2E-06
	segregation of mitotic sister chromatids		2E-06
	arrest in G2 phase		3E-06
	condensation of chromosomes		4E-06
	delay in initiation of M phase		4E-06
	prometaphase		5E-06
	arrest in G2/M phase transition of tumor cell lines		6E-06
tumor, cancer	female genital tract serous cancer		1E-10
	serous neoplasm		2E-09
	colon cancer		3E-08
	uterine serous papillary cancer		2E-07
	chromosomal congression of tumor cell lines		3E-07
	genital tract cancer		3E-07
	breast or ovarian cancer		5E-07
	ovarian cancer		5E-07
	pelvic tumor		8E-07
	pelvic cancer		1E-06
	breast or ovarian carcinoma		4E-06
	breast cancer		7E-06
	benign ovarian tumor		7E-06
	gonadal tumor		9E-06
	recurrent non-small-cell lung carcinoma		1E-05
	Fanconi's anemia		1E-05

Supplementary Table S2. Enriched Ingenuity Pathway Analysis IPA - Diseases and Biofunctions terms in primary keratinocytes overexpressing miR-203. The 609 differentially expressed probe sets after miR-203 overexpression in keratinocytes were subjected to functional enrichment analysis with IPA Disease and Biofunctions database. Terms with zscore >1,5 and pvalue<0.05 (Activation) or zscore<-1.5 and pvalue<0.05 (Inhibition) or pvalue<10E-5 (Modulation) are detailed.

Supplementary Table S3: PCR primer sequences.

abbrev (iHOP)	Amplicon's Size	Sense primer	Anti-sense primer
RPS9	431	GATGAGAAGGACCCACGGCGTCTGTTCG	GAGACAATCCAGCAGCCCAGGAGGGAC
RPL13A	483	TAAACAGGTACTGCTGGGCCGGAAGGTG	CACGTTCTTCTCGGCCTGTTTCCGTAGC
B2M	228	TTTCATCCATCCGACATTGA	CCTCCATGATGCTGCTTACA
SRC	142	GTGGCCATCAAAACCCTGAA	CCGTGACGATGTAAATGGGC
RAPGEF1	114	TCCGTAAAGATTCCAGAGAAG	GGTGGACATAAATTCTACTGC
MKI67	214	TGATAGCTTTACAAGCGCTCCAAAGC	CTTGGTTCCCGTGACGCTTCCATC
IVL	194	CTGGGAACAGCATGAGGAAT	GGAGCTCCAACAGTTGCTCT
LOR	213	CTACCTGGCCGTCCAAATAGATCC	GGAGGTAGTTGTACAGAAACCAAAGAG
CALML5	242	AACTACGAGGAGTTCGCGAGGATG	GCCCAAGGTCTGAAGGCAGAGAG
LCE1A	117	CCTGGGATCCAGAAACTTGA	GCAGGAAATGAGCTGGAGAG
FLG	215	TGATGGTATTCAAGTTGGCTCA	TGTTTCTCTTGGGCTCTTGG
SPRR1A	233	TCAGCAGCAGAAGCAGCCTTG	GTGCTGGAGTGACCGTTGAAGG
CDSN	430	GTGCCCAGGCATAGGGTTAGCTCAG	GCATATTGGGTGGGTTGACTAGATGTCG
